# Gossypol Treatment Restores Insufficient Apoptotic Function of DFF40/CAD in Human Glioblastoma Cells

**DOI:** 10.3390/cancers13215579

**Published:** 2021-11-08

**Authors:** Laura Martínez-Escardó, Montse Alemany, María Sánchez-Osuna, Alejandro Sánchez-Chardi, Meritxell Roig-Martínez, Salvio Suárez-García, Daniel Ruiz-Molina, Noemi Vidal, Gerard Plans, Carles Majós, Judit Ribas, María Antonia Baltrons, Jose R. Bayascas, Carlos Barcia, Jordi Bruna, Victor J. Yuste

**Affiliations:** 1 Cell Death, Senescence and Survival Group, Departament de Bioquímica i Biologia Molecular and Institut de Neurociències, Facultat de Medicina, Campus de Bellaterra, Universitat Autònoma de Barcelona, 08193 Bellaterra, Spain; laura.martinez.escardo@uab.cat (L.M.-E.); malemany@bellvitgehospital.cat (M.A.); maria.sanchez.osuna@umontreal.ca (M.S.-O.); MariaAntonia.Baltrons@uab.cat (M.A.B.); 2 Centro de Investigación Biomédica en Red Sobre Enfermedades Neurodegenerativas (C.I.B.E.R.N.E.D.), Campus de Bellaterra, 08193 Bellaterra, Spain; 3 Neuro-Oncology Unit, Hospital Universitari de Bellvitge-ICO L’Hospitalet (IDIBELL), 089098 Barcelona, Spain; nvidal@bellvitgehospital.cat (N.V.); gplans@bellvitgehospital.cat (G.P.); cmajos@bellvitgehospital.cat (C.M.); jbruna@bellvitgehospital.cat (J.B.); 4 Servei de Microscopia, Facultat de Biociències, Universitat Autònoma de Barcelona, 08193 Bellaterra, Spain; Alejandro.Sanchez.Chardi@uab.cat; 5 Neuroimmunity Research Group, Institut de Neurociències, Departament de Bioquímica i Biologia Molecular, Facultat de Biociències, Universitat Autònoma de Barcelona, 08193 Bellaterra, Spain; Meritxell.Roig@uab.cat (M.R.-M.); Carlos.Barcia@uab.cat (C.B.); 6 Catalan Institute of Nanoscience and Nanotechnology (ICN2), CSIC and BIST, 08193 Bellaterra, Spain; salvio.suarez@icn2.cat (S.S.-G.); dani.ruiz@icn2.cat (D.R.-M.); 7 Pharmacology of Cellular Stress Group, Pharmacology Unit, Departament de Medicina Experimental, Facultat de Medicina, Universitat de Lleida/IRBLleida, 25198 Lleida, Spain; judit.ribas@udl.cat; 8 Signalling in the Central Nervous System Group, Departament de Bioquímica i Biologia Molecular, Institut de Neurociències, Facultat de Medicina, Universitat Autònoma de Barcelona, 08193 Bellaterra, Spain; JoseRamon.Bayascas@uab.cat

**Keywords:** apoptosis, caspase-activated DNase (DFF40/CAD), glioblastoma (GBM), gossypol, nuclear fragmentation/disassembly

## Abstract

**Simple Summary:**

Human glioblastoma (GBM) cells are particularly resistant to nuclear fragmentation upon cytotoxic insult. To date, nuclear disassembly is the biological point-of-no-return of the apoptotic process. The injured cell that does not go beyond this point goes into a failed apoptosis and has potential for recovery. Nuclear disassembly is governed by caspases and, ultimately, by DFF40/CAD endonuclease. GBM cells express low levels of DFF40/CAD protein. Our aim was to assess whether this endonuclease could be activated to facilitate nuclear fragmentation in GBM cells. We revealed that GBM cells can activate their nuclear pool of DFF40/CAD in a caspase-dependent manner when treated with gossypol. Gossypol enabled DFF40/CAD assembly into high-order structures, facilitating nuclear dismantling. The identification of such compounds, pushing cells toward the point-of-no-return of apoptosis, will provide new tools to hamper the recovery of injured cells, slowing down tumor progression.

**Abstract:**

Glioblastoma (GBM) is a highly aggressive brain tumor and almost all patients die because of relapses. GBM-derived cells undergo cell death without nuclear fragmentation upon treatment with different apoptotic agents. Nuclear dismantling determines the point-of-no-return in the apoptotic process. DFF40/CAD is the main endonuclease implicated in apoptotic nuclear disassembly. To be properly activated, DFF40/CAD should reside in the cytosol. However, the endonuclease is poorly expressed in the cytosol and remains cumulated in the nucleus of GBM cells. Here, by employing commercial and non-commercial patient-derived GBM cells, we demonstrate that the natural terpenoid aldehyde gossypol prompts DFF40/CAD-dependent nuclear fragmentation. A comparative analysis between gossypol- and staurosporine-treated cells evidenced that levels of neither caspase activation nor DNA damage were correlated with the ability of each compound to induce nuclear fragmentation. Deconvoluted confocal images revealed that DFF40/CAD was almost completely excluded from the nucleus early after the staurosporine challenge. However, gossypol-treated cells maintained DFF40/CAD in the nucleus for longer times, shaping a ribbon-like structure piercing the nuclear fragments and building a network of bridged masses of compacted chromatin. Therefore, GBM cells can fragment their nuclei if treated with the adequate insult, making the cell death process irreversible.

## 1. Introduction

Glioblastoma (GBM) is the most common and lethal primary malignant brain tumor in adults. Almost all GBM-affected patients suffer from recurrences even after complete gross resection and radiochemotherapy [1]. During the last decade, a plethora of different approaches have been proposed for GBM treatment with scarce results [2]. A trait that contributes to GBM aggressiveness is its resistance to undergo apoptosis [3]. Apoptosis is a tightly regulated cell death program executed by a family of cysteine proteases, called caspases [4]. The most distinctive morphological alterations of apoptotic cells are chromatin condensation and nuclear fragmentation [5]. These nuclear changes are orchestrated by an endonuclease called DNA Fragmentation Factor, 40-kDa Subunit/Caspase-activated Deoxyribonuclease (DFF40/CAD) [6,7]. The activation of DFF40/CAD occurs in the cytosol, after the caspase-3-specific cleavage of its endogenous inhibitor, Inhibitor of Caspase-Activated DNase (ICAD) [6,8]. GBM cells express low levels of DFF40/CAD [9], and do not display fragmentation of the nuclei after exposure to different cytotoxic compounds [10]. Intriguingly, the overexpression of DFF40/CAD does not allow GBM cells to show those apoptotic nuclear alterations, even when caspases and ICAD are properly processed [9]. The endonuclease is mainly placed in the nucleus in either DFF40/CAD-overexpressing or wild-type GBM cells [9]. Therefore, it is reasonable to think that the cytotoxic compounds employed to date have not been proficient at activating the nuclear pool of DFF40/CAD. Given that, we asked whether the nuclear pool of DFF40/CAD could be activated to promote nuclear fragmentation in GBM cells.

Previously, we tested dozens of cytotoxic compounds to check their individual ability to promote or not apoptotic nuclear disassembly [10]. Since none of them promoted nuclear fragmentation in GBM cells, we pursued and tested other anticancer drugs. We used endoplasmic reticulum stressors (100 μM thapsigargin, 400 μM nordihydroguaiaretic acid, 100 μM apogossypol, 100 μM brefeldin A), antihistamines (20 μM astemizole, 20 μM terfenadine), BH3-mimetics (50 μM ABT-737, 100 μM gossypol, 100 μM sabutoclax, 100 μM obatoclax); kinase inhibitors (20 μM chelerytrine), and antilipemics (100 μM cerivastatin, 100 μM simvastatin) (data not shown). Here, we identify gossypol as a compound that increases the percentage of GBM cells displaying nuclear fragmentation. DFF40/CAD knockdown demonstrated that gossypol-induced apoptotic nuclear alterations relied on this endonuclease. A biochemical analysis of gossypol- and staurosporine-treated cells evidenced the activation of caspases and the processing of ICAD after both treatments. Deconvoluted confocal images revealed that gossypol allowed nuclear DFF40/CAD to form macromolecular complexes that facilitate the proper nuclear fragmentation of GBM cells.

## 2. Materials and Methods

### 2.1. Reagents and Cell Lines

The chemicals used in this study were gossypol (GSP) (Selleckchem, Houston, TX, USA; #S2303), pan-caspase inhibitor q-VD-OPh (qVD) (APExBIO, Boston, MA, USA; #A8165) and staurosporine (STP) (Merck KGaA, Darmstadt, Germany; #569397). Antibodies and cell lines used in this study are summarized in Tables S1 and S2, respectively.

### 2.2. Patient-Derived Non-Commercial GBM Cell Cultures

Patient-derived non-commercial GBM #04 cells (from first diagnosis) and #12 cells (from relapse) were isolated from tumor samples provided by Hospital Universitari de Bellvitge-ICO Duran i Reynals [9]. Once at 80–90% of confluence, GBM cells (including commercial cell lines) were treated with the corresponding compounds. Culture conditions are detailed in Supplementary Methods.

### 2.3. DEVD-Directed Activity

Quantitative DEVD-directed activity was performed as previously described [11] with minor modifications detailed in Supplementary Methods.

### 2.4. Immunofluorescence

Immunofluorescence of cells or paraffin-embedded tissue sections were performed as previously described [9,12] with minor modifications detailed in Supplementary Methods. Corrected Total Cell Fluorescence (CTCF) was calculated using the following formula: CTCF = Integrated Density − (Area of selected cell × Mean fluorescence of background readings) [13]. Values of the formula were obtained using Fiji-ImageJ software, version 2.1.0/1.53c (Bethesda, MD, USA). Intensity profile plots were obtained using the RGB profile plugging from Fiji-ImageJ software; see Supplementary Methods for details. Three-dimensional isosurface-rendered z-stack confocal images were generated using Imaris 8.3.4. (Bitplane AG, Zurich, Switzerland) software.

### 2.5. Lysosomes Labeling 

Lysosomes were stained with LysoTracker^TM^ Red DND-99 (Thermo Fisher Scientific, Waltham, MA, USA) following the manufacturer’s instructions with minor modifications detailed in Supplementary Methods.

### 2.6. Protein Extractions and Western Blotting

After the adequate treatments, protein lysates and Western blotting were performed as previously described [11]. For more details, see Supplementary Methods.

### 2.7. Transmission and Field Emission Scanning Electron Microscopy

#### 2.7.1. Transmission Electron Microscope (TEM) 

TEM was performed as previously described [10]. Details of the microscope and software employed are indicated in Supplementary Methods.

#### 2.7.2. Field Emission-Scanning Electron Microscopy (FE-SEM)

A total of 6 × 10^4^ cells were seeded onto circular coverglass (12 mm) (Thermo Fisher Scientific) placed into 24-well plate (Falcon^TM^, Thermo Fisher Scientific). After the adequate treatment, cells were fixed with 2.5% glutaraldehyde and 2% paraformaldehyde in 0.1 M phosphate buffer (PB) during 2 h at room temperature, rinsed 4 times in PB, post-fixed with 1% osmium tetroxide in PB during 1 h, and cryoprotected with graded series of sucrose (0.7, 1.5, and 0.3 M) during 3 h. Coverglasses were then sandwiched in metallic stubs, frozen in liquid nitrogen, and fractured. Samples were then dehydrated with graded series of ethanol, dried with CO_2_ in a Bal-Tec CPD030 critical-point dryer, mounted in metallic stubs, and observed without coating in a field emission-scanning electron microscope, ZEISS Merlin® FE-SEM, operating at 1 kV and equipped with a secondary electron detector.

### 2.8. Nuclear Morphology Analysis by Chromatin Staining

Nuclear morphology analysis was performed as previously described [14]. Briefly, cells were seeded in 96-well plates and fixed with 2% paraformaldehyde (*v*/*v*) (Electron microscopy sciences, Hatfield, PA, USA), permeabilized with 0.01% Triton (*v*/*v*), and stained with 1 μg/mL Hoechst 33,258 for 10 min at 4 °C after the indicated treatments. Nuclei were visualized under UV illumination with a Nikon ECLIPSE TE2000-E microscope equipped with epifluorescence optics and a Hamamatsu ORCA-ER photographic camera. More than 300 nuclei from each experimental condition from at least three independent experiments were scored.

### 2.9. DFF40/CAD Overexpression and Silencing

Cells were transfected with the eukaryotic expression vector pcDNA3.1, containing or not the open reading frame sequence of DFF40 (human CAD) (GenBank^TM^ accession number NM_004402) as previously described [10]. siRNA transfection was performed with DharmaFECT^TM^ (Thermo Fisher Scientific) as indicated by the manufacturer. The siRNAs sequences employed were 5′-GGAACAAGAUGGAAGAGAA-3′(hCAD-siRNA) [15] and 5′- AUAUGCGAUCGAGAUAUCG-3′ (NR-siRNA).

### 2.10. TUNEL Assay

LN-18 and SH-SY5Y cells were seeded onto 8-wells Lab-Tek chamber slides (Nunc^TM^ Lab-Tek^TM^ Chamber Slide System 177,445, Thermo Fisher Scientific). After the adequate treatment, detection of DNA fragments carrying 3′-OH groups was carried out as previously described [15]. Briefly, cells were fixed in freshly prepared 2% paraformaldehyde for 30 min at 4 °C, washed with PBS, and permeabilized with 0.1% Triton X-100 and 0.1% sodium citrate for 30–90 min at 4 °C. Then, cells were rinsed with PBS containing 0.1% Triton X-100 and incubated with 100 μL of a reaction mixture containing 0.025 nmol fluorescein-12-dUTP, 0.25 nmol dATP, 2.5 mM CoCl_2_, 40 units of recombinant terminal deoxynucleotidyl transferase, and 1X terminal deoxynucleotidyl transferase reaction buffer (Merck KGaA) for 1 h at 37 °C. The reaction was ended by adding 20 mM EGTA. Cells were washed twice with PBS, counterstained with 0.05 μg/mL Hoechst 33,258 in 20 mM EGTA, and microphotographs were taken under fluorescein isothiocyanate or UV filters in an epifluorescence microscope (Nikon ECLIPSE TE2000-E) coupled to a Hamamatsu ORCAER camera.

### 2.11. Statistical Analysis

For identify differences between experimental conditions, Student’s *t*-tests and one-way ANOVA test with Bonferroni correction as post hoc test were used. All calculations were performed using Package Prism–GraphPad Prism software, version 9.1.2 (San Diego, CA, USA). Hedges’ g and eta squared (η^2^) indicate effect size measures. *p* values < 0.05 were considered significant.

## 3. Results

### 3.1. Human Glioblastoma Cells Show Nuclear Alterations Compatible with Apoptotic Nuclear Morphologies When Challenged with Gossypol

**Figure 1:**
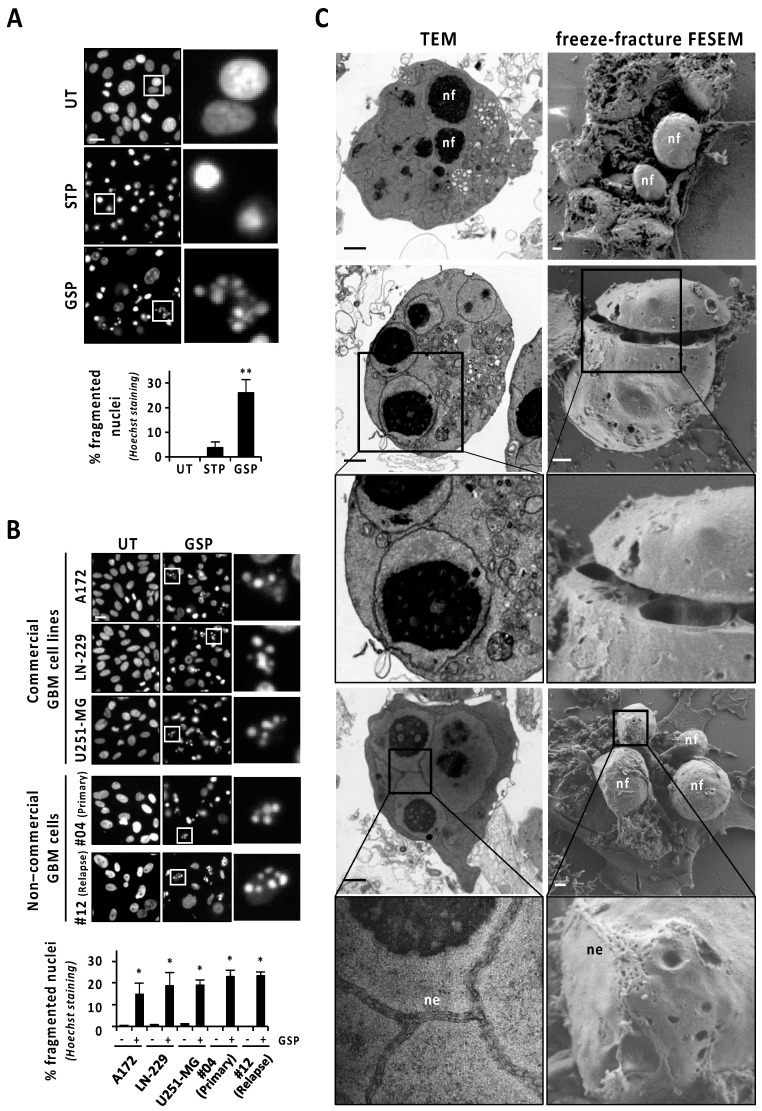
Gossypol induces nuclear fragmentation in human GBM cells. (**A**) LN-18 cells were treated with 1 μM staurosporine (STP) or 100 μM gossypol (GSP), or left untreated (UT). (**B**) Commercial and non-commercial (primary or relapse) GBM cells were treated with 100 µM gossypol, or left untreated. (**A**,**B**) After 24 h, cells were fixed and nuclei were stained with Hoechst 33258. Representative microphotographs of each condition are shown. The frames indicate the insets detailed in their respective right panels. Scale bars: 20 μm. The percentage of fragmented nuclei in each condition is represented by the mean ± SD (error bars). (**A**) η^2^ = 0.94. ** *p* < 0.001. (**B**) * *p* < 0.05. (**C**) LN-18 cells were treated with 100 μM gossypol for 14 h. Representative images of transmission electron microscopy (TEM) and freeze-fracture field emission-scanning electron microscopy (FESEM) showing nuclear fragments (nf) and nuclear envelope (ne). The frames indicate the inset detailed in the respective bottom panels. Scale bars: 1 µm.

Human glioblastoma (GBM)-derived cells hold an intrinsic defect at displaying canonical apoptotic nuclear morphologies, even when challenged with the classical apoptotic inducer staurosporine [10]. Nevertheless, we show here that the natural terpenoid aldehyde gossypol was able to increase the percentage of GBM cells displaying chromatin condensation and nuclear fragmentation. Gossypol was the only assayed compound inducing a significant percentage of cells with fragmented nuclei in LN-18 cells (26.02 ± 5.38%) (Figure 1A), a well-characterized human GBM-derived cell line [10]. The percentage of LN-18 cells showing fragmented nuclei after 24 h of staurosporine (3.75 ± 2.38%) was statistically non-significant compared with untreated cells (Figure 1A). As shown in Figure 1A, Hoechst staining revealed nuclei shrinkage and highly compacted chromatin in the absence of karyorrhexis as the main nuclear alterations in staurosporine-treated LN-18 cells. The concentration of gossypol inducing the highest percentage of cells showing fragmented nuclei was 100 μM. An increase or a reduction in this concentration resulted in less fragmented nuclei (Figure S1A). Starting from 9 h of gossypol treatment (100 μM), we detected a significant and progressive increase in nuclear fragmentation, sustained for at least 96 h (Figure S1B). The same concentration of gossypol induced nuclear fragmentation in other commercial GBM-derived cell lines (A172, LN-229, and U251-MG), and even in patient-derived non-commercial GBM cells, including samples from a primary GBM tumor (#04) and from a GBM relapse (#12) (Figure 1B). This result evidenced that all GBM cells tested possessed the intracellular machinery required to display nuclear disassembling. As shown in Figure 1C, transmission electron microscopy (TEM) analysis confirmed gossypol as a proficient stimulus to trigger alterations closely compatible with canonical apoptotic nuclear morphology. Those alterations were characterized by small pieces of highly packed round masses of condensed chromatin surrounded by a nuclear envelope. Likewise, freeze-fracture field emission-scanning electron microscopy (FE-SEM) was performed to obtain large, fractured planes of the plasma membrane and subcellular structures, which made the 3D ellipsoid structures characterized by a smooth drilled surface visible. These structures (nuclear fragments; nf in Figure 1C) were compatible in size and shape with those pieces of chromatin delimited by nuclear envelope observed by TEM. Interestingly, a nanoscale detail revealed that the nuclear envelope (ne in Figure 1C) shaped a zipper-like structure, observed by TEM and FE-SEM. These zipper-like organizations of the nuclear envelope could draw strips of nuclear breakage facilitating the complete disassembly of the nucleus (Figure 1C).

### 3.2. Caspases and DFF40/CAD Are Required for Gossypol-Induced Apoptotic Nuclear Morphologies

**Figure 2:**
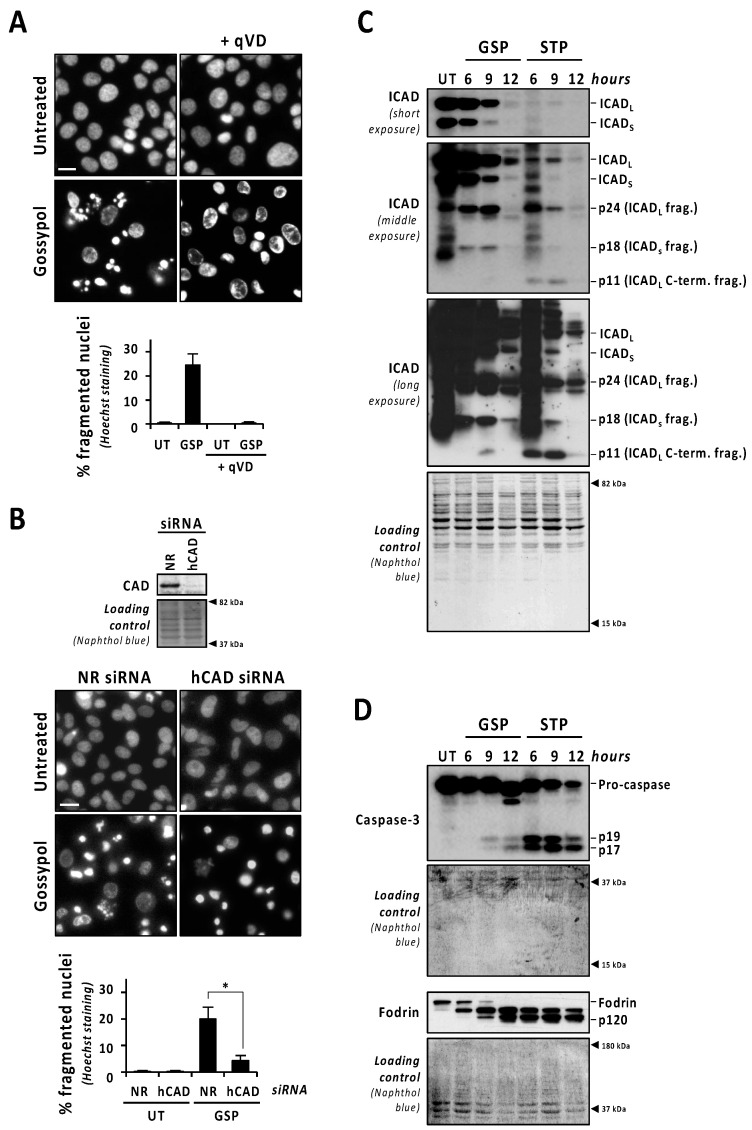
Gossypol induces the proper activation of the caspase-3/ICAD/CAD axis, although in a lesser extent than staurosporine. (**A**) LN-18 cells were left untreated (UT), or treated with 100 µM gossypol (GSP) in the presence (+) or in the absence of q-VD-OPh (qVD) (20 µM). (**B**) LN-18 cells were transfected with non-relevant (NR) or human CAD (hCAD) siRNA. After transfection, total protein extracts were obtained, and Western blotting confirmed the downregulation of DFF40/CAD. Then, cells were left untreated (UT) or treated with 100 µM gossypol (GSP). (**A**,**B**) After 24 h, cells were fixed and nuclei were stained with Hoechst 33,258. Representative microphotographs of each condition are shown. Scale bars: 20 μm. The percentage of fragmented nuclei in each condition is represented by the mean ± SD (error bars). (**B**) Hedges’ g = 2.6. * *p* < 0.05. (**C**,**D**) LN-18 cells were treated with 100 µM gossypol, 1 µM staurosporine, or left untreated (UT) for the indicated times. Then, cells were detached, Igepal-CA-630-soluble protein extracts were obtained, and Western blotting against ICAD (**C**), caspase-3, and α-fodrin (**D**) were performed. (**C**) The different caspase-mediated fragments of ICAD are indicated. (**D**) The detection of α-fodrin p120 fragment confirmed caspase-3 activation. Naphthol blue staining served as protein loading control.

To stablish a direct correlation between the activation of caspases and gossypol-induced nuclear fragmentation, we used the pan-caspase inhibitor q-VD-OPh. As shown in Figure 2A, the addition of q-VD-OPh to the culture media of gossypol-treated LN-18 cells impeded nuclear fragmentation. Q-VD-OPh also impaired nuclear dismantling in gossypol-treated patient-derived non-commercial GBM cells #04 and #12 (Figure S2). To ascertain the implication of DFF40/CAD in the nuclear alterations induced by gossypol, we proceeded with its silencing by a specific siRNA. We scored the percentage of fragmented nuclei induced by gossypol in non-relevant (NR) or human CAD (hCAD) siRNA-transfected LN-18 cells. As shown in Figure 2B, nuclear fragmentation was significantly impaired after the DFF40/CAD knockdown (4.21 ± 1.97%). Moreover, we analyzed the nuclear changes induced by gossypol in mock or DFF40/CAD overexpressing LN-18 cells. Although non-statistically significant, the percentage of fragmented nuclei was higher in DFF40/CAD-overexpressing (25.13 ± 4.55%) than in mock-transfected gossypol-treated cells (17.47 ± 0.18%) (Figure S3). Collectively, these results indicated that the intracellular machinery driving the final apoptotic nuclear outcome can be efficiently activated in GBM cells.

### 3.3. The Activation of Caspases and DFF40/CAD Are Necessary but Not Sufficient to Trigger Apoptotic Nuclear Disassembling after Gossypol Treatment

**Figure 3:**
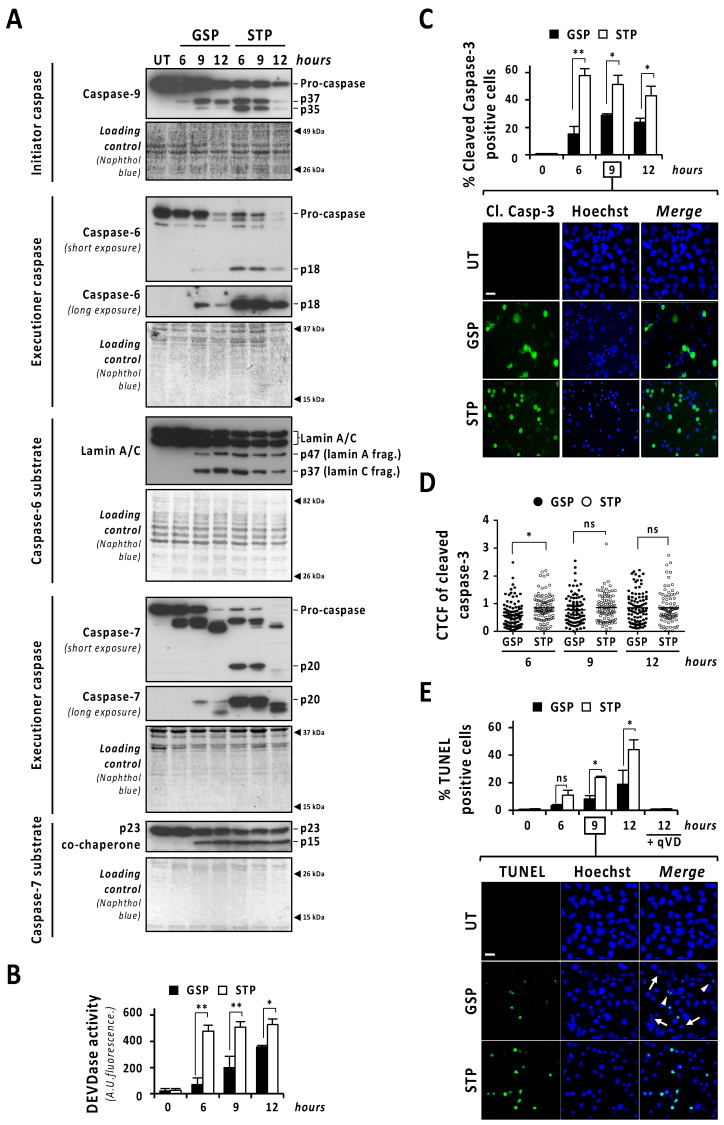
The percentage of cells showing caspase activation and TUNEL positivity is lower in gossypol- than in staurosporine-treated cells. LN-18 cells were treated with 100 µM gossypol (GSP) or 1 µM staurosporine (STP) for 6, 9 and 12 h, or left untreated (UT). (**A**,**B**) After treatment, cells were detached and protein extracts were obtained. (**A**) Western blotting against caspase-9, caspase-6 and caspase-7 were performed. The activation of caspase-6, and -7 was corroborated by the detection of p47 and p37 (lamin A/C) and p15 (p23 co-chaperone) proteolytic fragments, respectively. Naphthol blue staining served as protein loading control. (**B**) DEVDase activity was performed, and data obtained are expressed as the mean of arbitrary units (A.U.) of fluorescence ± SD. η^2^ = 0.97. (**C**,**D**) Immunofluorescence against cleaved caspase-3 (Cl. Casp-3) was performed. (**C**) The percentage of cleaved caspase-3-positive cells (green) is represented by the mean ± SD (error bars). (**C**) η^2^ = 0.96. (**D**) Corrected total cell fluorescence (CTCF) was calculated from different cleaved caspase-3-positive cells. CTCF value of each cell analyzed is represented as a dot in the scatter plot. The lines correspond to the mean ± SD (error bars). η^2^ = 0.04. (**E**) LN-18 cells were treated as indicated, in presence or absence of 20 µM q-VD-OPh (qVD) and TUNEL assay was performed. The percentage of TUNEL-positive cells (green) is represented by the mean ± SD (error bars). η^2^ = 0.95. (**C**,**E**) Representative images of 9 h of treatment are shown. Nuclei, stained with Hoechst 33,258, are shown in blue. Scale bars: 20 μm. Arrowheads indicate TUNEL-positive fragmented nuclei. Filled arrows indicate TUNEL-negative fragmented nuclei. (* *p* < 0.05 and ** *p* < 0.001).

The caspase-mediated processing of ICAD was analyzed by Western blot in a time-course of gossypol or staurosporine treatment. As shown in Figure 2C (low exposure panel), gossypol induced the progressive decrease in both ICAD_L_ (long) and ICADs (short) isoform expression levels, whereas staurosporine promoted an abrupt reduction as soon as 6 h after the beginning of the treatment. Surprisingly, the caspase-3 cleavage product p11 C-terminal fragment, which is considered an indicator of a complete release and activation of DFF40/CAD [16], was evident, starting from 6 h of staurosporine treatment, whereas it was barely detected after 9 h of gossypol (Figure 2C, middle and high exposure panels). Hence, levels of p11 fragment were disconnected from the quality of DFF40/CAD activation and the percentage of fragmented nuclei in GBM cells. As shown in Figure 2D, the cleavage of caspase-3 into its p19 and p17 fragments was initially detected at 6 h of staurosporine treatment, whereas it was barely evident at 9 h of gossypol. Likewise, staurosporine was more efficient than gossypol at inducing the cleavage of α-fodrin into its caspase-3-specific p120 fragment (Figure 2D). To corroborate the relevance of caspase-3 in gossypol-induced apoptotic nuclear fragmentation, we employed the caspase-3 null MCF-7 cell line. MCF-7 cells cultured in the presence of gossypol did not display apoptotic nuclear disassembly, correlating with the absence of the p11 fragment of ICAD (Figure S4). Moreover, staurosporine-treated LN-18 cells showed earlier and stronger processing and activation of both initiator caspase-9 and executioner caspases-6 and -7 than gossypol-treated cells (Figure 3A). Indeed, long exposures were needed to detect the cleaved forms p18 and p20 of caspase-6 and -7, respectively, after gossypol treatment. The activation of caspase-6 and -7 was corroborated by cleavage of lamin A/C into p47/p37 fragments [17], and p23 co-chaperone into p15 fragment [18], respectively (Figure 3A). According to these results, DEVDase-like activities were consistently higher in staurosporine- with respect to gossypol-treated cells at any time tested (Figure 3B). To ascertain if the differences between both compounds were attributable to different percentages of cells activating caspases, we performed immunofluorescences against cleaved caspase-3 at 6, 9, and 12 h of treatments. At any of the assayed times, the percentage of positive cells was lower when gossypol was applied (Figure 3C). Overall, these results indicated that the lower processing of caspases detected by Western blot was correlated with fewer cells activating caspases upon gossypol treatment. At that point, we wished to address whether the higher ability of gossypol to induce apoptotic nuclear morphologies could be the due to a higher number of activated caspases per cell. To address that question, we performed a single cell analysis of cleaved caspase-3-positive cells after 6, 9, and 12 h of gossypol or staurosporine treatment. After 6 h of treatment, the mean of the corrected total cell fluorescence (CTCF) for cleaved caspase-3 was higher in staurosporine- than in gossypol-treated cells. This difference was not further detected at later time points (Figure 3D). Altogether, the biochemical and biological analysis of the activation of caspases did not shed light on the ability of gossypol to provoke apoptotic nuclear morphologies in GBM cells.

### 3.4. Gossypol Induces Both Caspase-Dependent and Caspase-Independent DNA Damage

**Figure 4:**
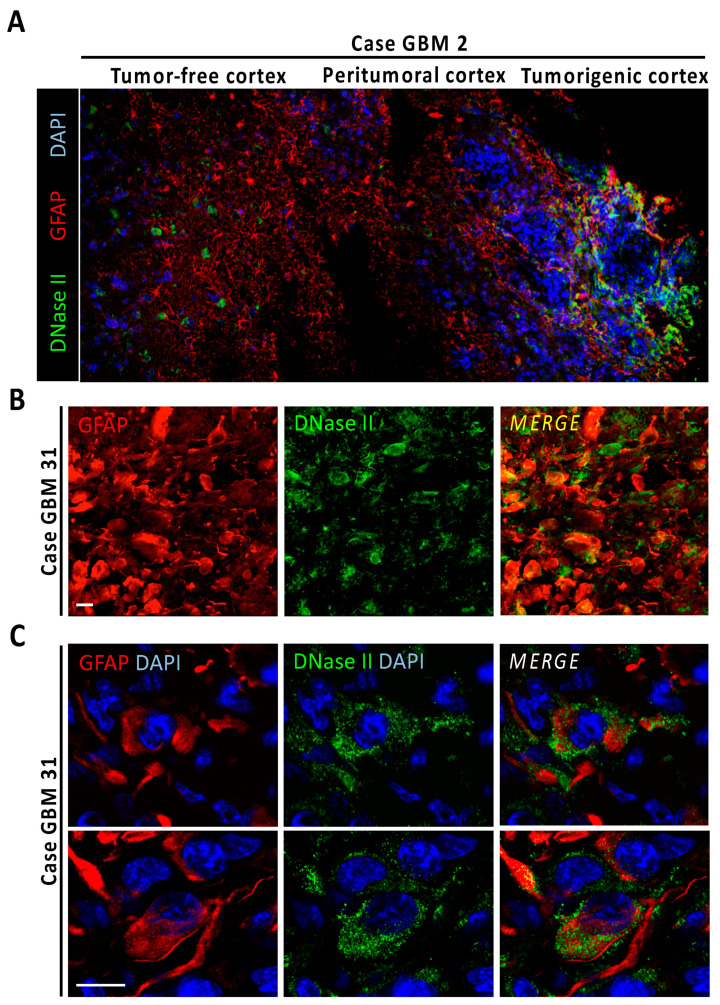
DNase-II expression in human GBM. (**A**) Fluorescence image mosaic from GBM biopsy (case GBM2) containing tumor-free cortex, peritumoral cortex, and tumorigenic tissue identified by high cellularity, evidenced by DAPI nuclear staining (blue) and high GFAP reactivity (red). Note the overexpression of DNase-II (green) at the tumor area. (**B**) Immunoreactivity of DNase-II in a human GBM case with GFAP-positive gemistocytic formations (case GBM31). (**C**) High resolution DNase-II-expressing cells from gemistocytic formations. Scale bars: 25 μm.

A standard readout of DFF40/CAD endonuclease activity is based on the detection of free 3′-OH DNA ends by TUNEL assay [19]. LN-18 cells treated with gossypol or staurosporine displayed less TUNEL reactivity than the apoptotic competent neuroblastoma-derived SH-SY5Y cells (Figure S5). A time-course of 12 h in LN-18 cells evidenced that gossypol consistently induced lower percentages of TUNEL-positive cells compared with staurosporine. The TUNEL positivity induced by any of the compounds was abolished in the presence of q-VD-OPh (Figure 3E). A detailed analysis of fragmented nuclei in gossypol-treated LN-18 cells revealed that 54.19 ± 3.17% of fragmented nuclei were TUNEL negative (Figure S5). By contrast, 9.01 ± 0.23% of fragmented nuclei were TUNEL negative after gossypol treatment in SH-SY5Y cells. This observation suggested a potential implication of other nucleases with the ability to generate DNA free-ends that cannot be detected by TUNEL assay in GBM. In that sense, lysosomal DNase-II is the main endonuclease generating 3′-P and 5′-OH DNA ends [20]. Accordingly, we focused our interest in the characterization of this DNase in a repository of inhouse human GBM biopsies. Samples were processed for immunohistofluorescence to detect glial fibrillary acidic protein (GFAP) in combination with anti-DNase-II antibodies, and DAPI as nuclear counterstaining. One of the samples, containing tumor and surrounding tumor-free tissue (case GBM2), enabled visualization of the regional differences regarding DNase-II expression. Pluricellular regions with high and polymorphic GFAP reactivity allowed the recognition of the tumorigenic cortex (Figure 4A). Peritumoral areas surrounding tumor nodes showed highly reactive protoplasmic GFAP-positive astrocytes, which were gradually reduced when approaching the tumor-free cortex (Figure 4A and Figure S7). In tumor-free and peritumoral areas, DNase-II was only expressed in cortical neurons, as previously described (The Human Protein Atlas, http://www.proteinatlas.org, accessed on 15 June 2021) [21], but not in GFAP-positive neighboring astrocytes (Figure 4A and Figure S7). However, and most importantly, DNase-II immunoreactivity was diffused and heterogeneously expressed in tumorigenic tissue. In this GBM case, DNase-II expression was seen both in glioma fibers and cell bodies (Figure S7). Detailed confocal analysis of tumor-free cortex with higher resolution settings revealed both cytoplasmic and nuclear DNase-II expression in cortical neurons, whereas neighboring astrocytes showed very low or absent expression (Figure S7). Conversely, higher resolution images of GFAP-positive tumorigenic cells displayed high DNase-II cytoplasmic expression with scarce nuclear staining (Figure S7). A tumor sample with characteristic gemistocytic formations (case GBM31), also displayed heterogeneous DNase-II expression at the tumor core (Figure 4B). High resolution images revealed that most of the globous gemistocytic cells showed DNase-II cytoplasmatic puncta with reduced nuclear expression (Figure 4C). A third case containing hypoxic pseudopalisades (case GBM34) also showed DNase-II expression in glioma cell bodies and, to a lower extent, in fibers (Figure S7).

**Figure 5:**
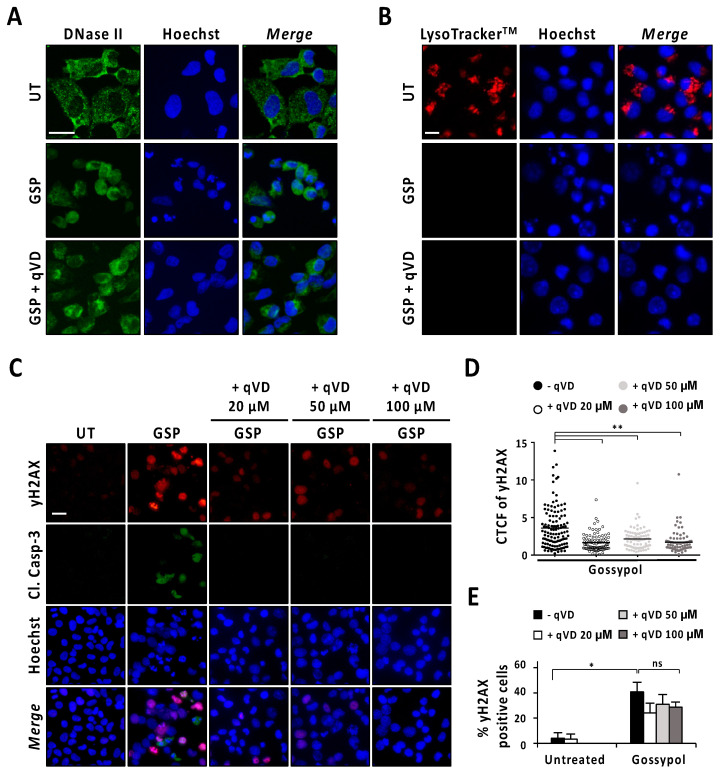
Analysis of DNase-II activation after gossypol treatment. (**A**,**B**) LN-18 cells were treated with 100 µM gossypol (GSP) in the presence (+) or absence of 20 µM q-VD-OPh (qVD) for 9 h, or left untreated (UT). (**A**) Cells were fixed and immunofluorescence against DNase-II (green) was performed. Nuclei were stained with Hoechst 33,258 (blue). (**B**) Lysotracker^TM^ (red) was added to the culture media for 30 min. The media were removed, and nuclei were stained with Hoechst 33,342 (blue). (**C**–**E**) LN-18 cells were left untreated (UT), or treated with 100 µM gossypol (GSP) in the presence (+) or absence of 20, 50 or 100 µM q-VD-OPh (qVD). After 9 h, cells were fixed and immunofluorescences against γH2AX (red) and cleaved caspase-3 (Cl. Casp-3) (green) were performed. Nuclei were stained with Hoechst 33,258 (blue). (**A**–**C**) Representative microphotographs of each condition are shown. Scale bars: 20 μm. (**D**) Corrected total cell fluorescence (CTCF) was calculated from different γH2AX-positive cells. CTCF value of each cell analyzed is represented as a dot in the scatter plot. The lines correspond to the mean. η^2^ = 0.16. (**E**) The percentage of γH2AX-positive cells is represented by the mean ± SD (error bars). η^2^ = 0.87. * *p* < 0.05 and ** *p* < 0.001. ns: not significant.

Next, we analyzed the intracellular distribution pattern of DNase-II by immunofluorescence staining in LN-18 cells after 9 h of gossypol treatment in the presence or absence of q-VD-OPh. As shown in Figure 5A, untreated cells presented a punctate distribution mainly located at the cytoplasm. By contrast, gossypol-treated cells showed a diffuse pattern of DNase-II staining that was also observed in LN-18 cells treated with gossypol in the presence of q-VD-OPh. In the same line, LysoTracker^TM^ evidenced lysosome disruption in gossypol-treated cells either in the presence or in the absence of q-VD-OPh (Figure 5B). The lysosome integrity was lost, starting at 1 h of gossypol treatment, regardless of the caspase activity status (Figure S8). Since lysosome integrity and the subcellular distribution pattern of DNase-II in gossypol-challenged cells were not affected by the presence of q-VD-OPh, we wondered whether cells treated with the combination of q-VD-OPh and the terpenoid underwent DNA damage. We used a phospho-specific antibody against the phosphorylation of H2AX at serine139 (γH2AX) to detect DNA breaks [22]. As shown in Figure 5C, cells treated with gossypol in the absence or presence of increasing concentrations of q-VD-OPh (20, 50, or 100 μM) presented γH2AX immunoreactivity. Notably, we detected a reduction in the fluorescence intensity of γH2AX in cells treated with gossypol and the pan-caspase inhibitor (Figure 5D). Irrespective of the reduction in fluorescence intensity, the percentage of γH2AX-positive cells induced by gossypol remained similar in the presence of any of the concentrations of q-VD-OPh employed (Figure 5E). By contrast, γH2AX immunoreactivity induced by staurosporine was prevented when LN-18 cells were co-treated with q-VD-OPh (Figure S9). These observations indicated that DNA damage was caspase-dependent in staurosporine-treated cells, and both caspase-dependent and -independent in gossypol-challenged cells. Taken all together, we cannot rule out the potential involvement of DNase-II in the apoptotic process triggered by gossypol. However, we dismiss its implication in DFF40/CAD-mediated nuclear disassembly since the inhibition of caspases did not noticeably affect the intracellular distribution pattern of DNase-II after gossypol treatment.

### 3.5. Gossypol Facilitates Nuclear DFF40/CAD Assembly into High-Order Structures

**Figure 6:**
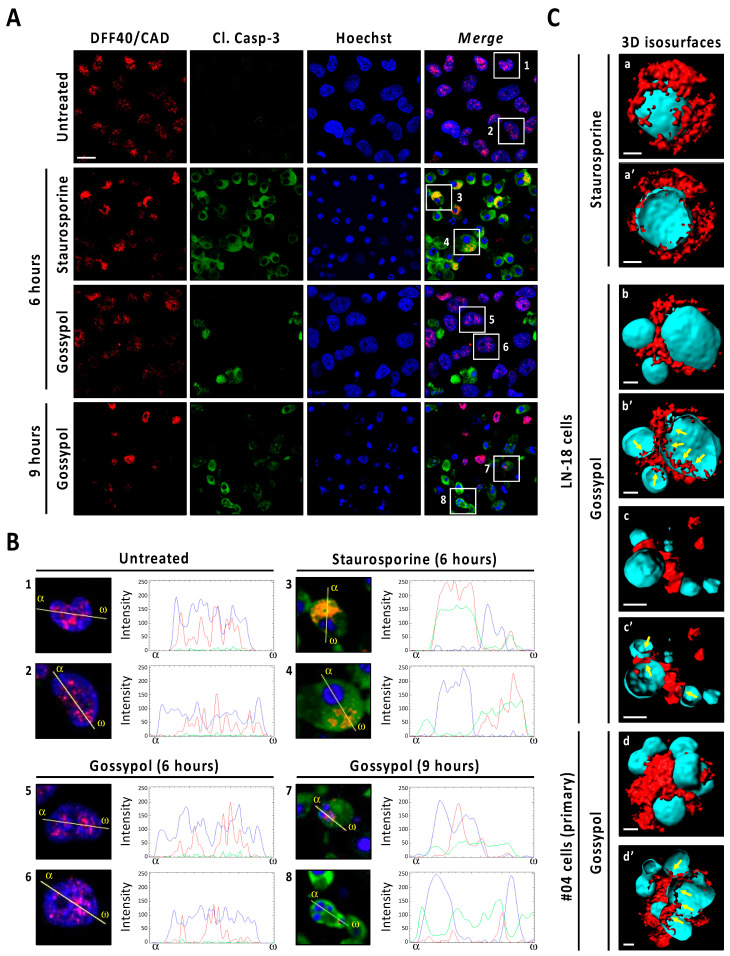
Immunofluorescence analysis of DFF40/CAD distribution in staurosporine- and gossypol-treated glioblastoma cells. (**A**,**B**) LN-18 cells were treated with 100 µM gossypol (GSP) or 1 µM staurosporine (STP), or left untreated (UT). After the indicated times, cells were fixed and immunofluorescences against DFF40/CAD (red) and cleaved caspase-3 (Cl. Casp-3) (green) were performed. Nuclei were stained with Hoechst 33,258 (blue). (**A**) Representative deconvoluted confocal scanning images of each condition are shown. Scale bar: 10 μm. (**B**) Higher magnifications of the cells framed in (**A**), and intensity profile plots showing the fluorescence distribution determined for sections of the cell indicated by the yellow line. α indicates the beginning (left side) of each profile plot. (**C**) 3D isosurface-rendered z-stack deconvoluted confocal images from individual commercial (LN-18) and non-commercial patient (#04)-derived GBM cells, treated as indicated (9 h), (a, b, c and d) were obtained with the 3D rendering software Imaris 8.3.4. (Bitplane). DFF40/CAD and nuclei 3D isosurface is represented in red and cyan color, respectively. Inside views (a’, b’, c’ and d’) were obtained using the clipping plane tool from the same software. Scale bars: 2 μm. Filled arrows indicate ribbon-like structures of DFF40/CAD inside the nucleus of gossypol-treated cells.

We proceeded with an in-depth characterization of DFF40/CAD subcellular localization after gossypol or staurosporine treatments by immunofluorescence. Deconvoluted confocal scanning images revealed a punctate pattern of DFF40/CAD immunoreactivity located at the nucleus of untreated cells (Figure 6A). While gossypol-challenged cells maintained the nuclear localization of DFF40/CAD, staurosporine-treated cells excluded the endonuclease from the nucleus as early as 6 h of treatment. In these cells, the line intensity profile indicated that DFF40/CAD was redistributed around the unique mass of highly condensed chromatin (Figure 6A). After 9 h, most of staurosporine-treated cells had lost DFF40/CAD immunoreactivity. The few cells exhibiting DFF40/CAD immunofluorescence did not show remarkable differences compared with those treated for 6 h (Figure S10). When analyzing cleaved caspase-3, most staurosporine-treated cells displayed a high immunoreactivity that correlated with high DFF40/CAD immunofluorescence at the cytosol (Figure 6A). Some cells also displayed cleaved caspase-3 but not DFF40/CAD immunoreactivity at the nucleus (Figure 6A). These observations were also evidenced in gossypol-treated cells. Differently, the nuclei of some gossypol-challenged cells presented DFF40/CAD immunofluorescence correlating with cleaved caspase-3 immunoreactivity (Figure 6B). The inside view of 3D isosurface rendered z-stack confocal images of staurosporine-challenged cells showed few spots of nuclear DFF40/CAD (Figure 6C). In the case of gossypol-treated cells we observed higher amounts of nuclear DFF40/CAD. Moreover, nuclear and cytosolic DFF40/CAD shaped a ribbon-like structure of a continuous surface connecting different small-sized masses of DNA in gossypol-treated cells. These features were also seen in patient-derived non-commercial GBM cells (Figure 6C). Altogether, these results suggested that each insult differentially distributes DFF40/CAD endonuclease, determining its final apoptotic function.

## 4. Discussion

Here, we demonstrate that GBM cells can activate their nuclear pool of DFF40/CAD to fragment the nucleus when stimulated by a proper trigger, such as gossypol. To our knowledge, gossypol is the only compound described to date that significantly rises the percentage of GBM cells showing nuclear fragmentation [10]. Staurosporine, one of the most potent apoptotic triggers, promotes nuclei shrinkage with highly compacted chromatin without signs of karyorrhexis in GBM cells [10]. It is broadly reported that this alkaloid fully activates the apoptotic program, provided that the challenged cells contain the required wild-type enzymatic apoptotic machinery. An alteration in the activation of caspases, or a dysfunction in the ICAD/CAD complex leads staurosporine-treated cells to undergo incomplete apoptotic cell death. MCF-7 cells, which are caspase-3 defective [23], or IMR-5, IMR-32, and GBM-derived cells, which express low levels of DFF40/CAD [9,24], are examples displaying unfinished apoptotic cell death after staurosporine treatment. To be fully activated, DFF40/CAD should reside in the cytosol, where caspase-3 and ICAD are mainly processed [25]. In GBM cells, DFF40/CAD is poorly detected in the cytosol [9]. This particular trait has been suggested as the main reason explaining the incomplete apoptosis observed in these cells after being exposed to different apoptotic agents. In this work, we have unraveled that DFF40/CAD located in the nucleus of healthy GBM cells can also be activated to facilitate nuclear disassembly upon gossypol treatment. This raises a question: why is staurosporine unable to prompt GBM cells to finish the apoptotic program? As presented here, staurosporine promotes an almost complete exclusion of DFF40/CAD towards the cytosol at earlier times of treatment than gossypol. In injured GBM cells, a timely retention of the nuclear DFF40/CAD could be key to build time for its activation at the nucleus. In that sense, DFF40/CAD will have enough time to assemble into high-order structures required to fragment the DNA [26], probably at certain chromatin-breakage sites [27], weakening the nuclear envelope and governing the constriction of the nucleus to facilitate its disassembly. The nucleolytic action of DFF40/CAD could reduce the strength that results from chromatin attachments (matrix/scaffold attachment regions, MARs/SARs) to the nuclear matrix [28,29]. Then, it is likely that DFF40/CAD in the nucleus of healthy cells could act as a tag of specific potential susceptible regions to distortion, resembling the process described for nuclear envelope breakdown during mitosis [30].

To ensure the proper function of tissues and organs, cells without a functional purpose will be eliminated and the remaining empty area replenished by clonal expansion of neighboring cells. The targeted killing will be executed by cellular competition, in which a cell population eliminates another by inducing its death [31,32]. To replenish the empty space, neighboring cells have to be skilled to interpret the signals sent by dying cells. The way a cell dies could be pivotal for neighboring cells to interpret the signals released as survival/proliferation or toxic. On theoretical grounds, GBM cells should hold an adaptive behavior to read and interpret the molecular signals specifically released from an unfinished apoptotic cell death on its own behalf. Then, it is tempting to speculate that changing the way GBM cells die could provoke an impairment to the surrounding surviving cells to decode the signals released as survival/proliferation instructions. A better comprehension of the relationship between the subroutine of cell death engaged and the tumor cell response will enable us to better predict the effectiveness of a therapeutic approach. Since DFF40/CAD also acts as a gatekeeper of genomic stability, a deeper knowledge of its activation could be of capital relevance to slow the progression of genomic instability and plasticity, impairing the environment adaptability of tumor cells [33,34,35,36]. In this line, given that GBM cells hold an intrinsic deficiency in the expression of DFF40/CAD, we may speculate that these cells may be programmed either to not activate, or to activate, the endonuclease in a sublethal mode. A suboptimal activation of DFF40/CAD due to a sublethal activity of caspases entails mutagenesis [37,38,39], which could counteract the adverse extracellular environment provoked by the chemotherapeutic employed.

In summary, gossypol is presented here as an insult which unveils new cytotoxic responses in GBM cells. We demonstrate that GBM cells, which poorly retain DFF40/CAD at the cytosol [9,10], can activate their nuclear pool after gossypol treatment. The activation of the nuclear pool of DFF40/CAD is sufficient to promote apoptotic nuclear disassembly. A better understanding of the biological consequences derived from a proper activation of DFF40/CAD could help to improve the management of GBM patients, which still remains a huge challenge for scientists and clinicians.

## 5. Conclusions

We show that, despite low expression levels of DFF40/CAD, GBM cells can display apoptotic nuclear disassembly. The nuclear pool of the endonuclease can be properly activated, as long as GBM cells are challenged with the appropriate trigger. Among the compounds tested, gossypol was the only one causing such an effect. We confirm that gossypol is a good activator of DFF40/CAD in a caspase-dependent manner. This alkaloid grants a timely nuclear retention of the endonuclease to build time for its activation in the nucleus of GBM cells. DFF40/CAD activated in gossypol-treated cells assembles into high-order structures that are required to fragment the DNA.

We have taken a step forward in the comprehension of the intimate molecular mechanisms governing the latest stages of apoptosis, particularly those orchestrated by DFF40/CAD. Unravelling that GBM cells can enter into the point-of-no-return of apoptosis entails that those cells have no opportunity to acquire adaptive advantages. This finding is of special interest regarding the aggressiveness of GBM and its refractory nature towards the current therapeutic strategies. Indeed, improving the efficacy of the compounds potentially employed to manage this illness, in terms of apoptosis induction, could be paramount to tackle its malignant progression. Overall, the data presented in this manuscript suggest that the proper activation of harmful molecular pathways disabled by tumor cells could be a tempting strategy to better manage GBM patients.

## Data Availability

The data presented in this study are available on request from the corresponding author.

## Supplementary Materials

The following are available online at https://www.mdpi.com/article/10.3390/cancers13215579/s1, Figure S1: Gossypol-induced nuclear fragmentation is dose- and time-dependent, Figure S2: The pan-caspase inhibitor q-VD-OPh impairs gossypol-induced nuclear fragmentation in patient-derived non-commercial GBM cells, Figure S3: DFF40/CAD overexpression does not significantly alter/modify the percentage of gossypol-induced apoptotic nuclear morphologies, Figure S4: Gossypol does not induce apoptotic nuclear disassembling in caspase-3-null MCF-7 cells, Figure S5: TUNEL reactivity in LN-18 cells is lower than in SH-SY5Y cells regardless of the insult employed. Figure S6: LN-18 cells show higher percentage of TUNEL-negative fragmented nuclei than SH-SY5Y cells after gossypol challenge, Figure S7: DNase-II expression in human GBM, Figure S8: The addition of q-VD-OPh does not impede gossypol-triggered lysosome damage, Figure S9: The phosphorylation of H2AX induced by staurosporine is recued in the presence of q-VD-OPh, Figure S10: DFF40/CAD is excluded from the nuclei in staurosporine-treated glioblastoma cells, Table S1: Antibodies used in this study, Table S2: Human Cell Lines used in this study, Table S3: Clinical data from glioblastoma patients of sections used for DNase II/GFAP immunohistofluorescences. Supplementary methods are also included.
