# Supplementary material for: Gossypol Treatment Restores Insufficient Apoptotic Function of DFF40/CAD in Human Glioblastoma Cells
Source: Cancers (Basel). 2021 Nov 8;13(21):5579. doi: 10.3390/cancers13215579 (PMC8583586; doi:10.3390/cancers13215579)
Supplement: Supplementary file 1 [file cancers-13-05579-s001.zip › cancers-1387738 supplementary.pdf]

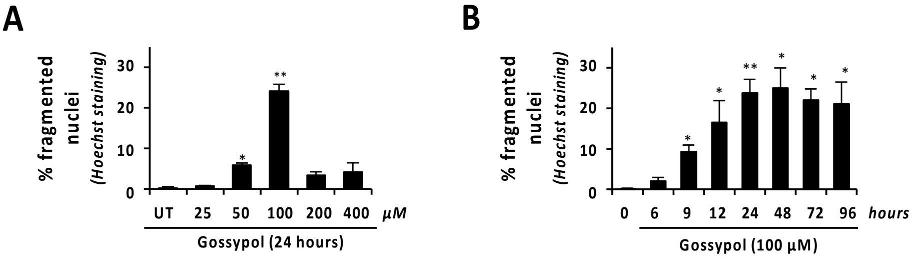

Figure S1. Gossypol-induced fragmented nuclei are dose- and time-dependent. (**A**) LN-18 cells were treated with different concentrations of gossypol ranging from 25 to 400 μM for 24 h, or left untreated (UT). (**B**) LN-18 cells were treated with 100 μM gossypol for 6, 9, 12, 24, 48, 72, and 96 h, or left untreated. (**A**, **B**) After treatment, nuclear morphology was assessed by fixing cells and staining the nuclei with Hoechst 33258. Fragmented nuclei were scored and the corresponding percentages are represented by the mean ± SD (error bars). (**A**) $\eta^2 = 0.99$. (**B**) $\eta^2 = 0.93$. $*$ $p < 0.05$ and $**$ $p < 0.001$.

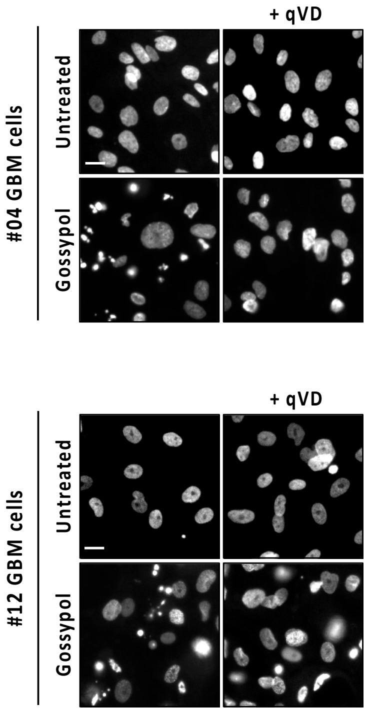

Figure S2. The pan-caspase inhibitor q-VD-OPh impairs gossypol-induced nuclear fragmentation in patient-derived non-commercial GBM cells. Patient-derived non-commercial #04 and #12 GBM cells were left untreated (UT), or treated with 100 μM gossypol (GSP) in the presence (+) or in the absence of q-VD-OPh (qVD) (20 μM). After 24 h, cells were fixed and nuclei were stained with Hoechst 33258. Representative microphotographs of each condition are shown. Scale bars: 20 μm.

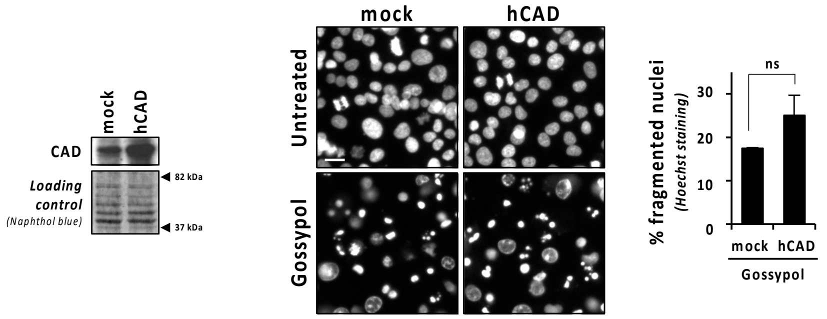

Figure S3. DFF40/CAD overexpression does not significantly alter/modify the percentage of gossypol-induced apoptotic nuclear morphologies. LN-18 cells were stably transfected with the human ORF of DFF40/CAD (hCAD) (NM_004402) or with the empty vector (mock). Total protein was extracted from untreated cells, and Western blotting against DFF40/CAD was performed to corroborate the overexpression of the endonuclease. Naphthol blue staining served as protein loading control. Then, cells were left untreated or treated with 100 μM gossypol for 24 h. After treatment, cells were fixed and nuclear morphology was analyzed by staining the nuclei with Hoechst 33258. Representative microphotographs of each condition are shown. The percentage of fragmented nuclei was calculated and the graph represents the mean ± SD (error bars). Hedges' g = 1.36. ns: not significant. Scale bar: 20 μm.

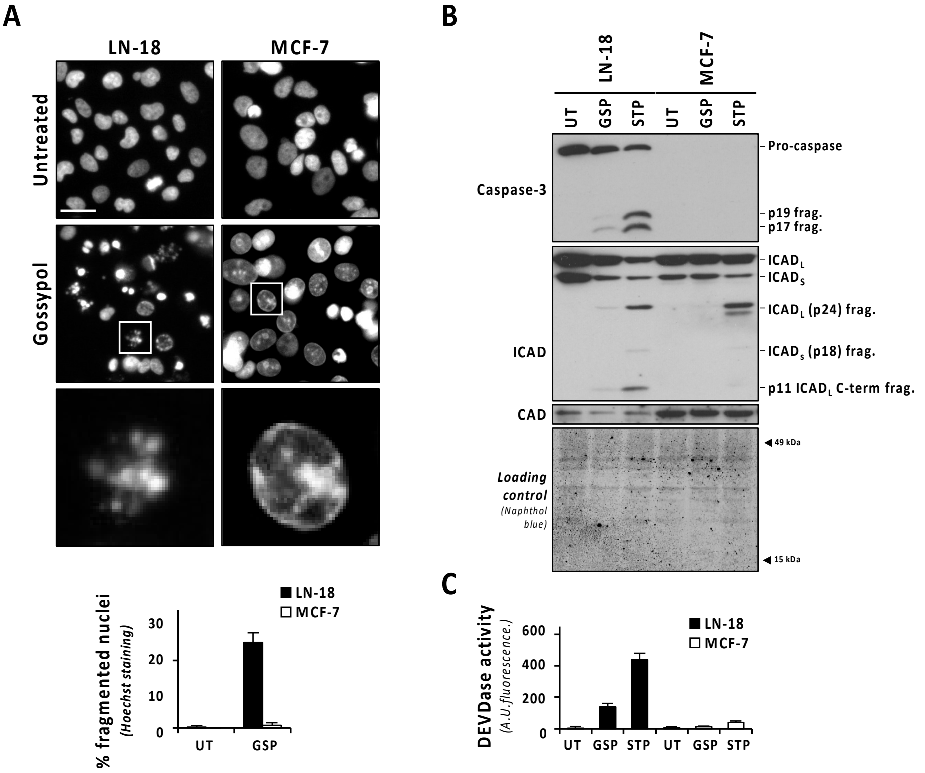

Figure S4. Gossypol do not induce apoptotic nuclear disassembling in caspase-3-null MCF-7 cells. (**A–C**) MCF-7 and LN-18 cells were treated with 100 μM gossypol (GSP), or left untreated (UT). (**A**) After 24 h of treatment, nuclear morphology was assessed by fixing cells and staining the nuclei with Hoechst 33258. The percentage of fragmented nuclei was calculated and the graph represents the mean ± SD (error bars). The frames indicate the inset detailed in their respective bottom panels. Scale bar: 20 μm. (**B–C**) After 9 h, cells were detached and protein extracts were obtained. LN-18 cells treated with 1 μM staurosporine (STP) were used as positive controls. (**B**) Western blotting against caspase-3, ICAD and CAD were performed. Naphthol blue staining served as protein loading control. (**C**) DEVDase activity was performed and data obtained are expressed as the mean of arbitrary units (A.U.) of fluorescence ± SD.

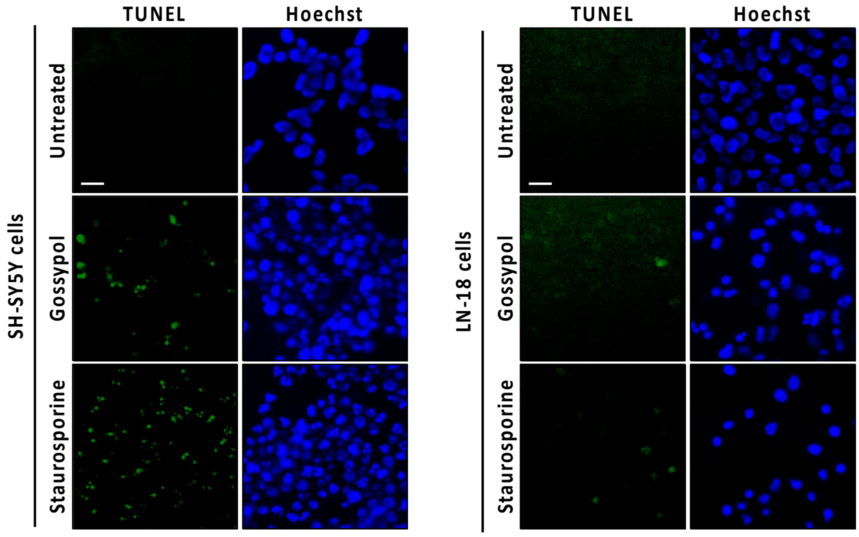

Figure S5. TUNEL reactivity in LN-18 cells is lower than in SH-SY5Y cells regardless of the insult employed**.** SH-SY5Y and LN18 cells were treated with 100 µM gossypol or 1 µM staurosporine for 9 h, or left untreated (UT). After treatment, TUNEL assay was performed. TUNEL-positive cells are shown in green. Nuclei, stained with Hoechst 33258, are shown in blue. Scale bar: 20 µm.

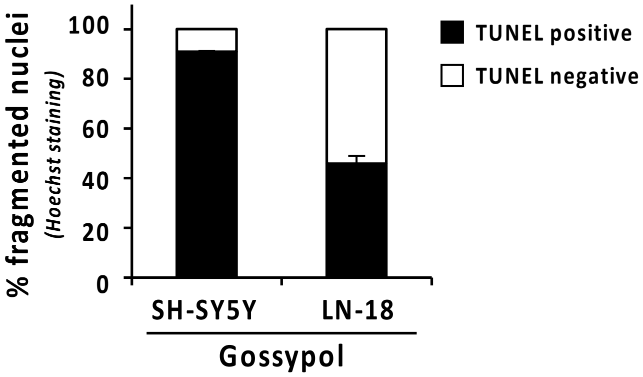

Figure S6. LN-18 cells show higher percentage of TUNEL negative fragmented nuclei than SH-SY5Y cells after gossypol challenge. SH-SY5Y and LN18 cells were left untreated (UT) or treated with 100 µM gossypol for 9 h. After treatment, TUNEL assay was performed. Nuclei were stained with Hoechst 33,258. Fragmented nuclei were scored as TUNEL-positive or TUNEL-negative, and the resulting percentages are represented by the mean ± SD (error bars).

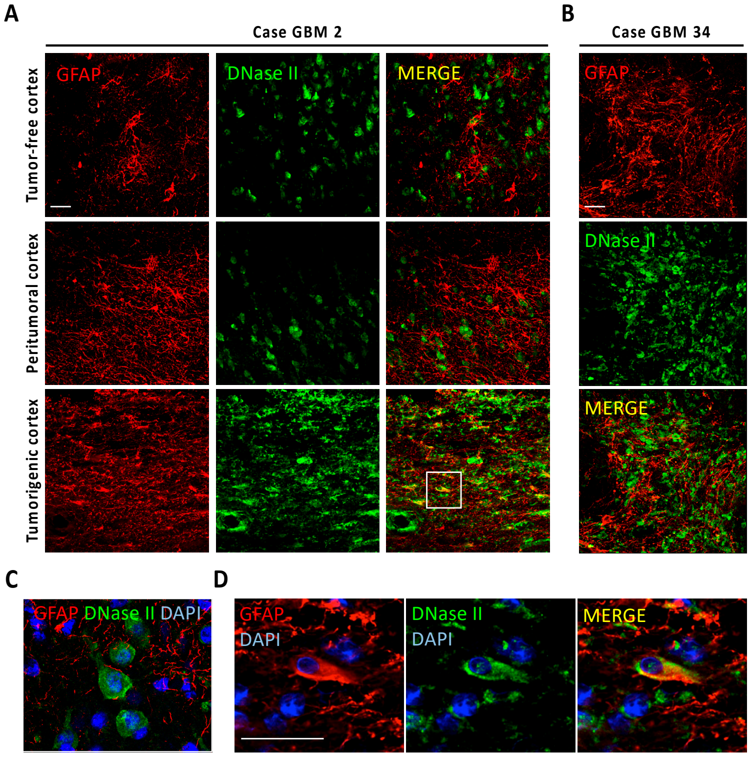

Figure S7. DNase-II expression in human GBM. (**A**) Higher magnification confocal imaging of human GBM sample (Case GBM 2) displayed in Figure 4. In tumor-free areas, DNase-II is highly expressed in cortical neurons. Neighboring GFAP-positive astrocytes are also depicted. In peritumoral cortex, the DNase-II expression is still appreciated in neurons but surrounded by highly reactive GFAP astrocytes. In tumorigenic cortex, DNase-II immuno-reactivity becomes highly heterogeneous, being expressed by both tumorigenic GFAP fibers and cell bodies. (**B**) Immunoreactivity of DNase-II in peri-necrotic areas of a pseudo-palisade rich tumor, potentially macrophages (Case GBM 34) (**C**) Detail of cortical neurons expressing DNase-II but absent in GFAP-positive neighboring astrocytes in tumor-free cortex (Case GBM 2). (**D**) Detail of DNase-II high expression in tumorigenic cortex from inset in A. Scale bars: 40 µm.

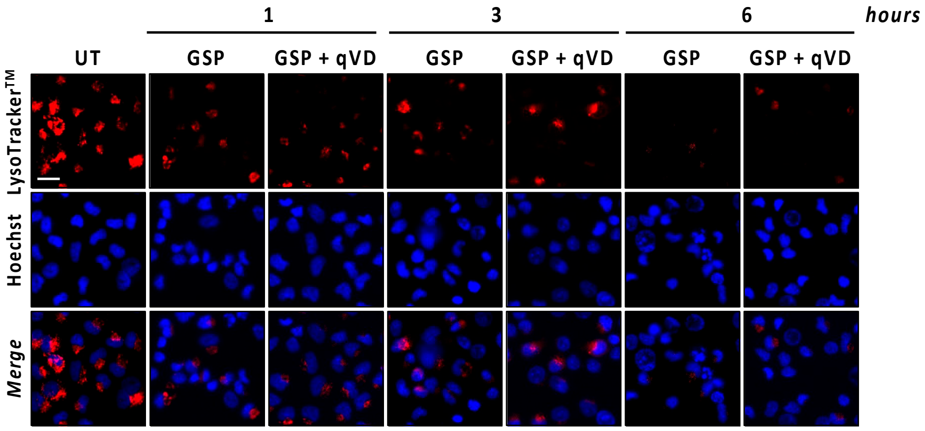

Figure S8. The addition of q-VD-OPh does not impede gossypol-triggered lysosome damage. LN-18 cells were left untreated (UT), or treated with 100 µM gossypol (GSP) in the presence (+) or absence of 20 µM q-VD-OPh (qVD) for the indicated times of treatment. After treatment, LysoTracker™ Red DND-99 was added to the culture media for 30 min. Prior to visualization, the media was removed and nuclei were stained with Hoechst 33342 (blue). Representative images of each condition are shown. Scale bar: 40 µm.

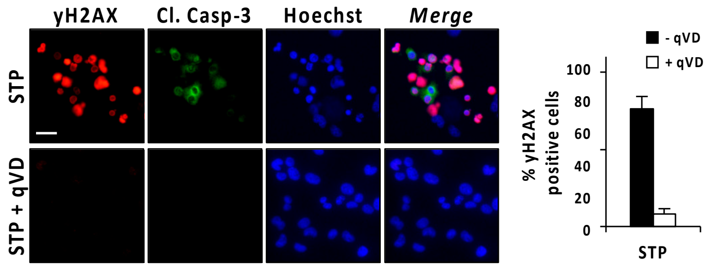

Figure S9. The phosphorylation of H2AX induced by staurosporine is recued in the presence of q-VD-OPh**.** LN-18 cells were treated with 1 μM staurosporine (STP) in the presence (+) or absence of 20 μM q-VD-OPh (qVD). After 9 h of treatment, cells were fixed and immunofluorescence against γH2AX (red) and cleaved caspase-3 (Cl. Casp-3) (green) was performed. Nuclei were stained with Hoechst 33258 (blue). Representative images of each condition are shown. Scale bar: 20 μm. The percentage of γH2AX positive cells is represented by the mean ± SD (error bars).

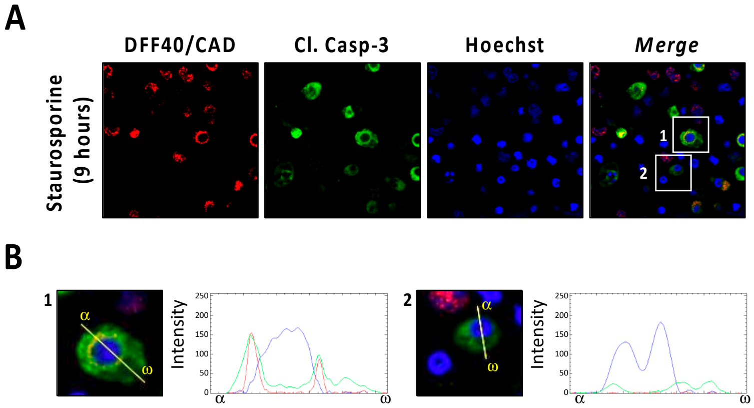

Figure S10. DFF40/CAD is excluded from the nuclei in staurosporine-treated glioblastoma cells**.** LN-18 cells were treated with 1 μM staurosporine (STP) for 9 h. Then, cells were fixed and immunofluorescence against DFF40/CAD (red) and cleaved caspase-3 (Cl. Casp-3) (green) was performed. Nuclei were stained with Hoechst 33258 (blue). (**A**) Representative deconvoluted confocal scanning images of each condition are shown. Scale bar: 10 μm. (**B**) Higher magnifications of the cells framed in A, and intensity profile plots showing the fluorescence distribution determined for sections of the cell indicated by the yellow line. α indicates the beginning (left side) of each profile plot.

Martínez-Escardó et al. Supplementary Methods.

Cell Lines and Culture procedures

Cell lines used in this study are summarized in Table S2.

All cells were routinely grown in 100-mm culture dishes (Flacon™ 353003, Fisher Scientific) containing 10 mL Dulbecco's modified Eagle's medium (DMEM) supplemented with 100 U/mL penicillin, 100 µg/mL streptomycin, and 10% heat-inactivated fetal bovine serum (FBS) (Invitrogen). Cells were maintained at 37 °C in a saturating humidity atmosphere containing 95% air and 5% CO_2. Once at 80–90% of confluence, cells were rinsed with phosphate-buffered saline (PBS) (100 mM pH 7.4) and incubated at 37 °C for 3 min with 0.05% trypsin-EDTA (Sigma-Aldrich) until their dissociation. Trypsin was neutralized by adding DMEM with 10% FBS, and the resulting cell suspension was centrifuged at 200 x g for 5 min. Finally, cell pellets were resuspended in complete medium. For the different experiments, cells were seeded at the adequate cell densities in culture dishes or multiwell plates (Falcon™, Fisher Scientific) using the same culture conditions as described above. All procedures were performed in a laminar flow cell culture hood using sterile techniques. Cells were routinely tested for contamination, including mycoplasma, by the Servei de Cultius Cel·lulars from Institut de Neurociències - UAB.

DEVD-directed Activity

Quantitative DEVD-directed activity in cell lysates was measured after incubating 20 µg of Igepal-CA-630-soluble protein extracts with 20 µM Ac-DEVD- aminofluoromethyl-coumarin (AFC) (Calbiochem) for 10 h at 35°C in 96-multiwell microplates. The assay was performed with a final volume of 100 µL/well containing 50 µL of 2X assay buffer (100 mM Tris-HCl pH 7.4, 4 mM EGTA, 10 mM DTT, 20% sucrose, 2 mM PMSF, 40 µM Ac-DEVD-AFC). Fluorescence intensity was obtained by using a BIO-TEK Synergy HT fluorometer under an excitation filter of 360 nm (40-nm bandwidth) and an emission filter of 530 nm (25-nm bandwidth). Results are expressed as the means of arbitrary units (A.U.) of fluorescence.

Immunofluorescence

0.03×10^6 cells were seeded onto 8-wells Lab-Tek chamber slides (Thermo Scientific™ Nunc™ Lab-Tek™ Chamber Slide System 177445, Fisher Scientific). After the indicated treatments, cells were fixed with 2% paraformaldehyde (v/v) (overnight at 4°C), washed once with PBS, and washed twice with PBS containing 0.1% Triton X-100 (v/v). Then, cells were preincubated for 30 min at 25°C with a blocking solution (3% bovine serum albumin (BSA) and 0.1% Triton X-100 in PBS), and incubated overnight at 4°C with the specific antibodies in blocking solution. After incubating primary antibodies, cells were washed three times with PBS containing 0.1% Triton X-100. Then, appropriate secondary antibodies were incubated for 45 min at 25°C in the presence of 0.5 µg/mL of Hoechst 33258 in blocking solution. Finally, chambers were removed, and slides were mounted with an aqueous mounting medium (FluorSaveTM reagent, Calbiochem).

Confocal images were acquired using a laser confocal microscope (Zeiss LSM 700, Carl Zeiss) and the specific confocal software (ZEN 2010, Carl Zeiss). Deconvoluted images were obtained by using the Huygens Essential (version 14.10.2, Scientific Volume Imaging, Hilversum, The Netherlands). Epifluorescence images were obtained with a Nikon ECLIPSE 90i microscope equipped with a Nikon DXM1200F photographic camera and ACT-1 software.

Fluorescence image mosaic from GBM biopsy (case GBM 2) was a composite of 36 microphotographs, 12 in each color, acquired using a laser confocal microscope (Zeiss LSM 700, Carl Zeiss), and assembled using the Stitching plugging from Image J software.

Image analysis

Intensity profile plots. Intensity profile plots were obtained by using the RGB profiler plugging from Fiji-ImageJ software (version 2.1.0/1.53c). First, the corresponding images were transformed to RGB color image (Image > Type > RGB color). Then, different lines were drawn and, finally, the RGB profiler plugging was used to obtain the red, green and blue profile plot for each line selection (Plugins > RGB profiler).

Lysosomes labeling

Cells were seeded in 96-multiwell plate at 0.01 x 10^6 cells/well. After the indicated treatments, 75 nM of LysoTracker™ Red DND-99 (Thermo Fisher Scientific) were added to the culture media for 30 min at 37 °C. Then, the culture media was removed and nuclei were stained with 2 μg/mL Hoechst 33342 for 10 min at 4 °C. Cells were visualized and photographed with a Nikon ECLIPSE TE2000-E microscope equipped with epifluorescence optics and a Hamamatsu ORCA-ER photographic camera.

Protein Extractions and Western Blotting

0.8 x 10^6 cells were seeded onto 60-mm culture dish (Falcon™, Fisher Scientific). After the adequate treatment, cells were detached, pelleted at 500 x g for 5 min, and washed once with PBS. Then, cells were lysed on ice for 15 min with 50 μl of Igepal buffer (50 mM Tris-HCl, pH 6.8, 1 mM EDTA, 150 mM NaCl, 1% Igepal CA-630 X-100, 1 x protease inhibitor cocktail (Roche Applied Science)). The supernatants were clarified by centrifugation at 21,000 x g for 10 min at 4°C. Alternatively, total extracts were obtained by lysing the cells with SET buffer (10 mM Tris-HCl, pH 6.8, 150 mM NaCl, 1 mM EDTA, 1% SDS) and heating the samples at 95°C for 10 min. In both cases, protein concentration was quantified by Lowry assay (DC protein assay, Bio-Rad) in 96-well plates. First, Reagen A and Reagen S were mixed in a ratio 50:1 and added at a volume of 25 μL/well. Second, 1 μL/well of standards or protein samples (triplicates) were added and, finally, mixed with 200 μL/well of Reagen B. Microplates were incubated at room temperature for a minimum of 15 min. Absorbance at 750 nm was measured with a Power Wave XS spectrophotometer (BioTek).

For Western blotting, 20–50 μg of protein were loaded in SDS-polyacrylamide gels. Protein samples were electrophoresed and electrotransferred onto nitrocellulose membranes (Protran Nitrocellulose Transfer membrane, GE Healthcare). After blocking with Tris-buffered saline (TBS), 0.1% Tween-20 (referred to as TBS-T) containing 5% nonfat dry milk, the membranes were probed with the appropriate specific primary antibodies for 1 h at room temperature or overnight at 4 °C. Note that primary antibodies were prepared in TBS-T. Then, membranes were washed 3 times with TBS 0.1% Tween-20 and incubated with the adequate secondary antibodies conjugated with peroxidase for 1 h at RT. Finally, immunoblots were developed with the ClarityTM Western ECL Blotting Substrate kit (Bio-Rad). The signals were developed by a film processor (Fujifilm FPM-100A) using X-ray films (Fujifilm Super RX-N), and scanned with a commercial scanner. Alternatively, signal was detected with the ChemiDoc™ MP Image System with Image Lab™ Software (BIO-RAD). Adjustments of brightness, contrast or color balance were applied to the entire image.

After the specific antibodies were blotted, membranes were stained for 5 min in a 10% methanol, 2% acetic acid solution containing 0.1% Naphthol blue. Then, membranes were destained in the 10% methanol and 2% acetic acid solution for 10 min, allowed to dry, scanned, and used as a loading control. The purpose of using Naphthol blue as loading control was to take profit of the entire lane to demonstrate that the total loading is similar, independently of the particular changes provoked by the different treatments. In this sense, it is important to take into account that during cellular dismantling, there are many proteins suffering cleavages or degradation. It is well known that cytoskeletal proteins, such as actin or tubulin [1,2], or glycolytic enzymes, such as GAPDH [3], are substrates of caspases. Hence, the use of those specific markers as loading controls to study cell death processes is discouraged.

Primary and secondary antibodies are listed in Table S1.

Transmission Electron Microscopy (TEM)

For transmission electron microscope pellets were fixed with 2.5% glutaraldehyde (Merck) and 2% paraformaldehyde (TAAB) in 0.1 M phosphate buffer (PB, Sigma-Aldrich) during 2 h, rinsed 4 times in PB, post-fixed in 1% osmium tetroxide (TAAB) containing 0.8% potassium hexoferrocyanide (Merck) in PB during 1 h, dehydrated in graded series of acetone, embedded in Epon resin (Ted Pella), and polymerized at 60 °C during 48 h. Ultrathin sections (70 nm) of selected areas from semithin sections (1 μm) were obtained with an ultramicrotome EM UC7 (Leica Microsystems) and contrasted following routine protocol of uranyl acetate and lead citrate solutions. Randomly distributed sections of at least 2 grids of each sample were analyzed in a TEM JEM 1400 (Jeol) operating at 80kV and equipped with an Erlangshen ES1000W CCD camera (Gatan).

Reference

1. Mashima, T.; Naito, M.; Noguchi, K.; Miller, D.K.; Nicholson, D.W.; Tsuruo, T. Actin cleavage by CPP-32/apopain during the development of apoptosis. *Oncogene* **1997**, *14*, 1007-1012, doi:10.1038/sj.onc.1200919.
2. Gerner, C.; Fröhwein, U.; Gotzmann, J.; Bayer, E.; Gelbmann, D.; Bursch, W.; Schulte-Hermann, R. The Fas-induced Apoptosis Analyzed by High Throughput Proteome Analysis. *Journal of Biological Chemistry* **2000**, *275*, 39018-39026, doi:10.1074/jbc.M006495200.
3. Shao, W.; Yeretssian, G.; Doiron, K.; Hussain, S.N.; Saleh, M. The Caspase-1 Digestome Identifies the Glycolysis Pathway as a Target during Infection and Septic Shock. *Journal of Biological Chemistry* **2007**, *282*, 36321-36329, doi:10.1074/jbc.M708182200.

Martínez-Escardó et al. Supplementary Tables

Table S1. Antibodies used in this study.

Use	Antibody	Source	Dilution	Cat. No.	Supplier
Western blot	Caspase-3	Rabbit	1:2,000	9662	Cell Signaling Technology
	Caspase-6 (clone 3E8)	Mouse	1:2,000	M070-3	MBL
	Caspase-7	Rabbit	1:2,000	9492	Cell Signaling Technology
	Caspase-9	Mouse	1:1,000	9508	Cell Signaling Technology
	DFF40/CAD	Rabbit	1:500	AB16926	Millipore Iberica S.A.U
	DFF45/ICAD (clone 6B8)	Mouse	1: 5,000	M037-3	MBL
	HRP-conjugated anti-mouse IgG	Rabbit	1:20,000	A9044	Sigma-Aldrich-
	HRP-conjugated anti-rabbit IgG	Goat	1:20,000	A0545	Sigma-Aldrich-
	Lamin A/C (clone JOL2)	Mouse	1:2,000	ab40567	Abcam
	p23 antibody (clone JJ3)	Mouse	1:10,000	NB300-576	Novus Biological Europe, Inc
	Spectrin α-chain (clone AA6)	Mouse	1:20,000	MAB1622	Millipore Iberica S.A.U
Immunofluorescence	Alexa Fluor488 goat anti-rabbit IgG	Goat	1:500	A11034	Thermo Fisher Scientific
	Alexa Fluor555 goat anti-mouse IgG	Goat	1:500	A21424	Thermo Fisher Scientific
	Alexa Fluor555 goat anti-chicken IgY	Goat	1:1,000	A21437	Thermo Fisher Scientific
	CAD (F11)	Mouse	1:40	sc-374067	Santa Cruz Biotechnology
	Cleaved caspase-3 (Asp175)	Rabbit	1:200	9661	Cell Signaling Technology
	DNase II	Rabbit	1:200	ab115233	Abcam
	GFAP	Chicken	1:200	ab4674	Abcam
	Phospho-histone H2A.X (Ser 139) (clone JBW301)	Mouse	1:2,000	05-636	Millipore Iberica S.A.U

Table S2. Human Cell Lines used in this study.

	Tumor type	Cell Line reference	Year of acquisition
**A172**	Glioblastoma	ATCC® CRL-1620™	2010
**LN-18**	Glioblastoma	ATCC® CRL-2610™	2010
**LN-229**	Glioblastoma	ATCC® CRL-2611™	2010
**MCF-7**	Breast adenocarcinoma	ATCC® HTB-22™	2002
**SH-SY5Y**	Neuroblastoma	ATCC® CRL-2266™	2009
**U251-MG**	Glioblastoma	ECACC 09063001	2013

Table S3. Clinical data from glioblastoma patients of sections used for DNase II / GFAP immunohistofluorescences.

	Sex	Glioma grade	Location	Ki67 index (%)	Vimentin	GV	PP	Aberrant mitoses
**Case GBM 2**	F	4	Right temporal - frontal	20	NR	++	NR	+
**Case GBM 31**	M	4	Left temporal	30	++	+++	++	+++
**Case GBM 34**	F	4	Left parietal	20	+++	++	+++	+

F, female; M, male; GV, glomeruloid vessels; PP, pseudopalisades; NR, not reported on medical record.
