# Supplementary material for: Gossypol Treatment Restores Insufficient Apoptotic Function of DFF40/CAD in Human Glioblastoma Cells
Source: Cancers (Basel). 2021 Nov 8;13(21):5579. doi: 10.3390/cancers13215579 (PMC8583586; doi:10.3390/cancers13215579)
Supplement: Supplementary file 1 [file cancers-13-05579-s001.zip › WB .pdf]

“Original Images for Blots/Gels”

Fig. 2B → CAD and loading control (naphthol blue):

Lanes from left to right inside the squared area: non-relevant (NR) and human CAD (hCAD) siRNA LN18-transfected cells.

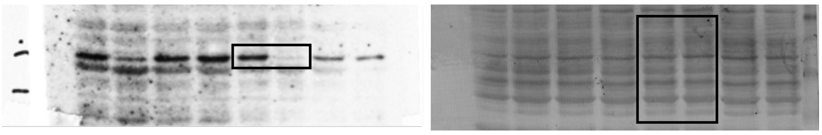

Fig. 2C → ICAD (short, middle and long exposures) and loading control (naphthol blue)

Lanes from left to right: Untreated (UT), gossypol (GSP) 6 hours, GSP 9 hours, GSP 12 hours, staurosporine (STP) 6 hours, STP 9 hours, STP 12 hours.

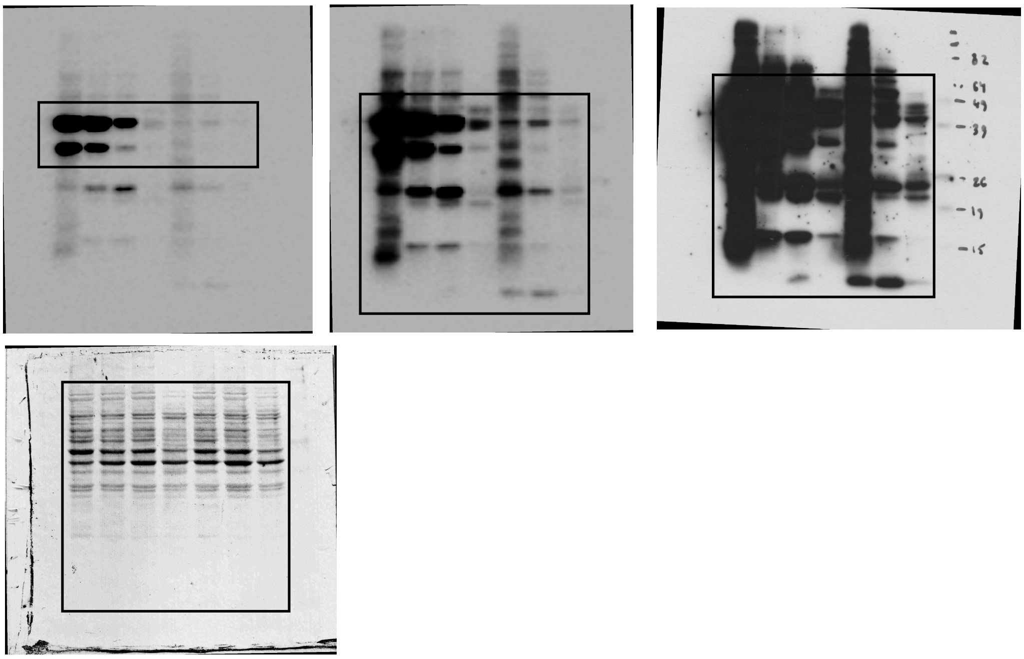

<u>Fig. 2D → Caspase-3 (short, middle and long exposures) and loading control (naphthol blue):</u>

Lanes from left to right: Untreated (UT), gossypol (GSP) 6 hours, GSP 9 hours, GSP 12 hours, staurosporine (STP) 6 hours, STP 9 hours, STP 12 hours.

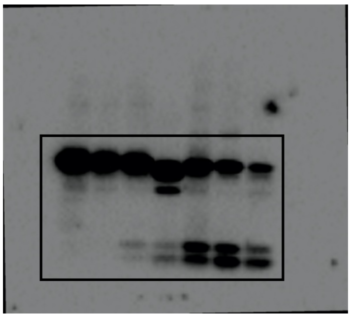

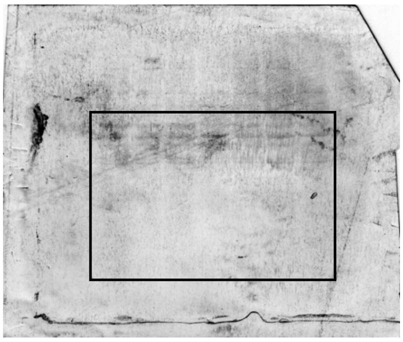

<u>Fig. 2D → Fodrin and loading control (naphthol blue):</u>

Lanes from left to right inside the squared area: Untreated (UT), gossypol (GSP) 6 hours, GSP 9 hours, GSP 12 hours, staurosporine (STP) 6 hours, STP 9 hours, STP 12 hours.

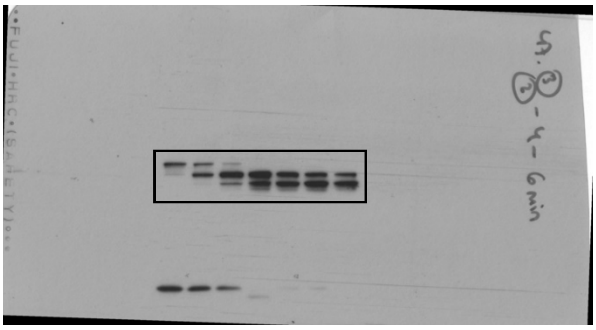

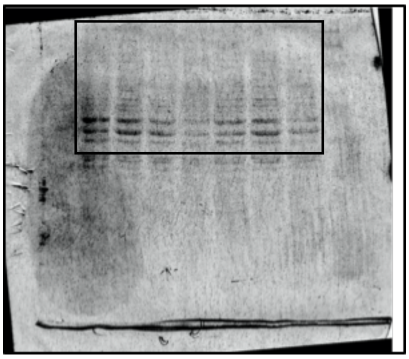

<u>**Fig. 3A → Caspase-9 and loading control (naphthol blue):**</u>

Lanes from left to right inside the squared area: Untreated (UT), gossypol (GSP) 6 hours, GSP 9 hours, GSP 12 hours, staurosporine (STP) 6 hours, STP 9 hours, STP 12 hours.

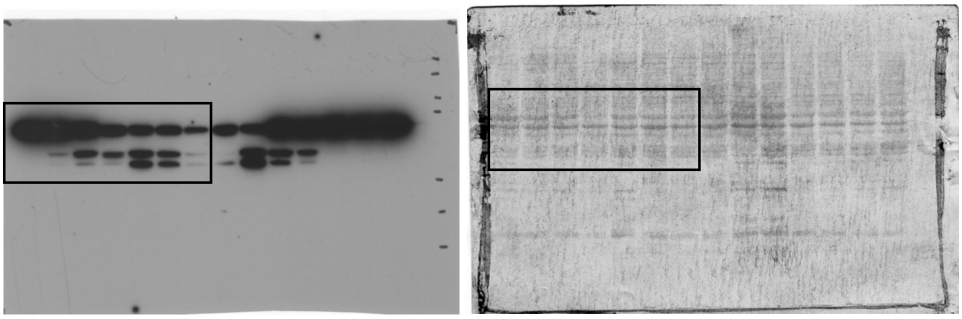

<u>**Fig. 3A → Caspase-6 (short and long exposures) and loading control (naphthol blue):**</u>

Lanes from left to right: Untreated (UT), gossypol (GSP) 6 hours, GSP 9 hours, GSP 12 hours, staurosporine (STP) 6 hours, STP 9 hours, STP 12 hours.

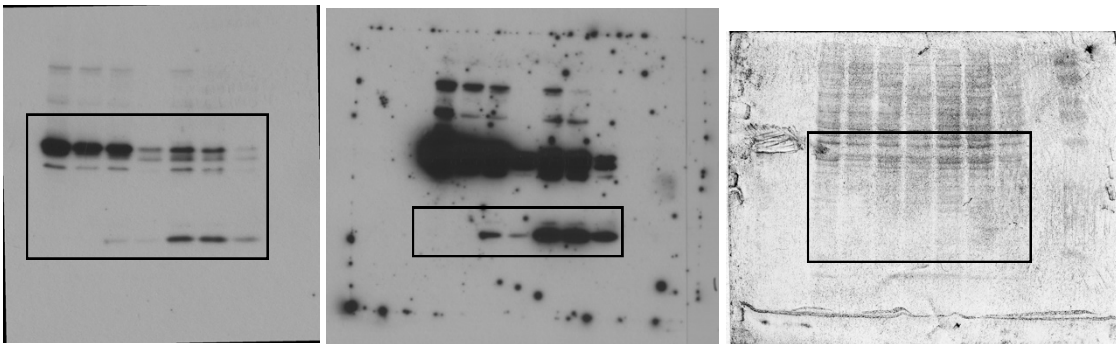

Fig. 3A → Lamin A/C and loading control (naphthol blue):

Lanes from left to right: Untreated (UT), gossypol (GSP) 6 hours, GSP 9 hours, GSP 12 hours, staurosporine (STP) 6 hours, STP 9 hours, STP 12 hours.

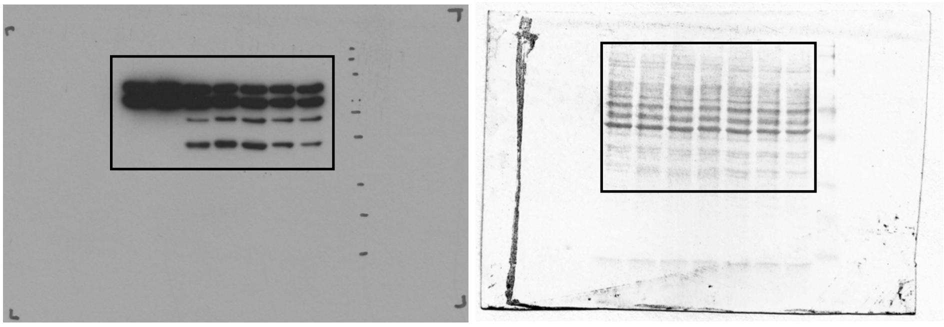

Fig. 3A → p23 and loading control (naphthol blue):

Lanes from left to right: Untreated (UT), gossypol (GSP) 6 hours, GSP 9 hours, GSP 12 hours, staurosporine (STP) 6 hours, STP 9 hours, STP 12 hours.

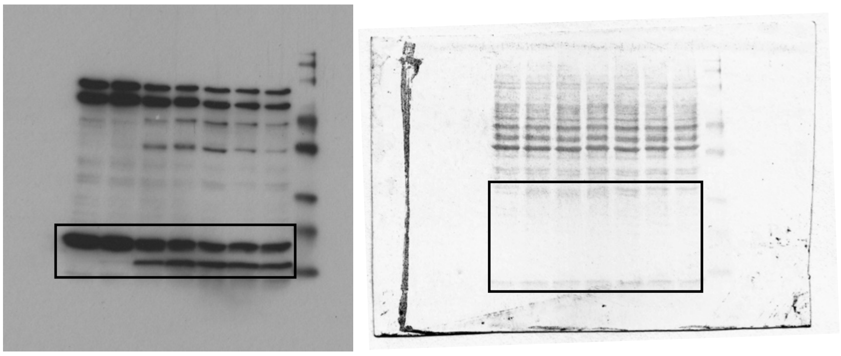

Fig. 3A → Caspase-7 (short and long exposures) and loading control (naphthol blue):

Lanes from left to right: Untreated (UT), gossypol (GSP) 6 hours, GSP 9 hours, GSP 12 hours, staurosporine (STP) 6 hours, STP 9 hours, STP 12 hours.

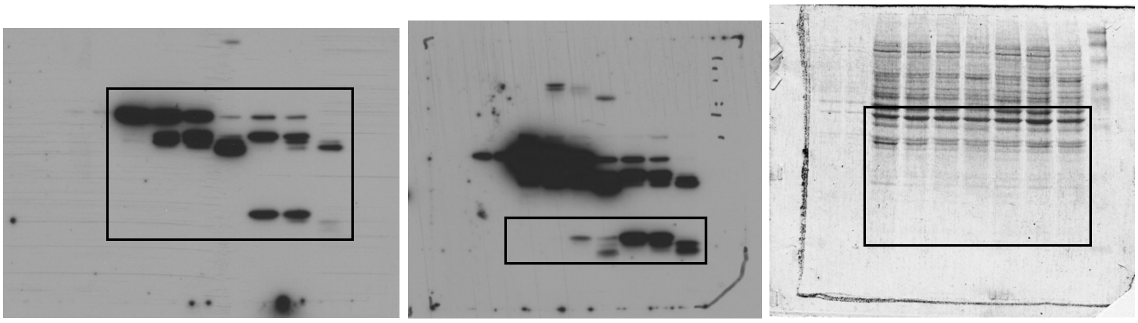

Supplementary Figures

Figure S2 → CAD and loading control (naphthol blue):

Lanes from left to right inside the squared area: LN18 pcDNA3 cells, LN18 pcDNA3/hCAD overexpressing cells.

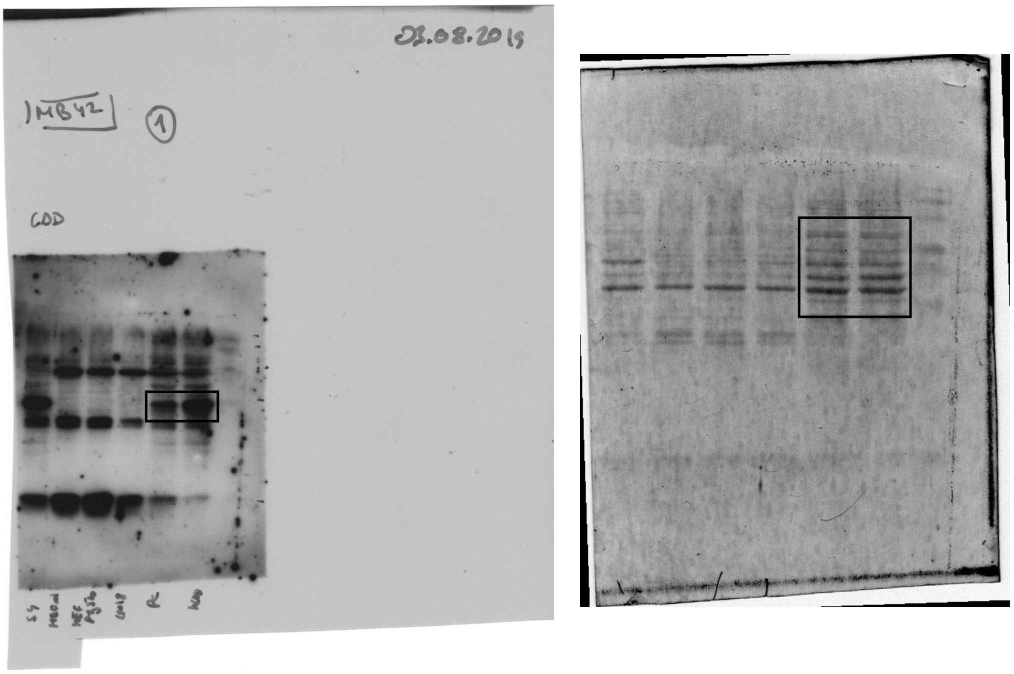

Figure S3 → Caspase-3, ICAD, CAD and loading control (naphthol blue):

Lanes from left to right inside the squared area: staurosporine (STP) LN-18 treated cells, Untreated (UT) MCF-7 cells, Gossypol (GSP) MCF-7 cells

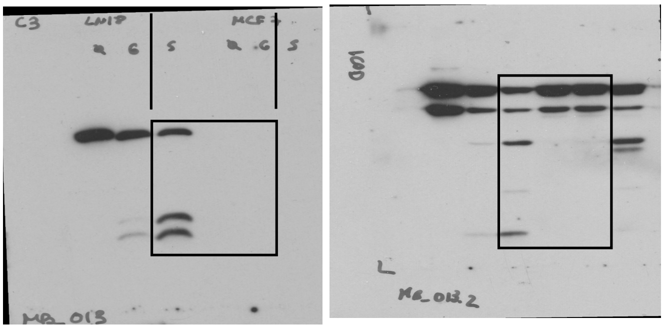

(Caspase-3) (ICAD)

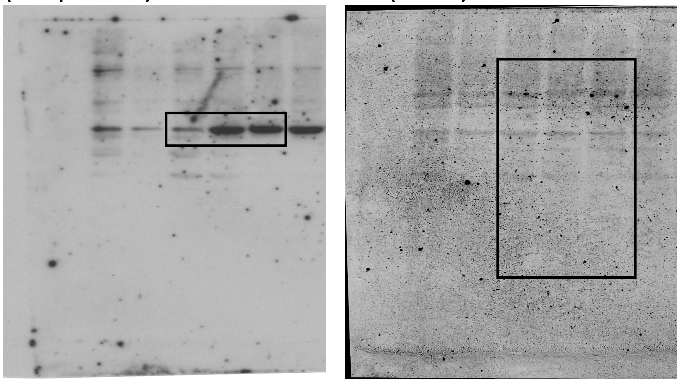

(CAD) (Loading control)
